# Global network and local vulnerabilities underlie brain atrophy across Parkinson’s disease stages

**DOI:** 10.1093/brain/awaf432

**Published:** 2025-11-14

**Authors:** Andrew Vo, Christina Tremblay, Shady Rahayel, Sarah Al-Bachari, Henk W Berendse, Joanna K Bright, Fernando Cendes, Emile d'Angremont, John C Dalrymple-Alford, Ines Debove, Michiel F Dirkx, Jason Druzgal, Gaëtan Garraux, Rick C Helmich, Michele T Hu, Neda Jahanshad, Martin E Johansson, Johannes C Klein, Max A Laansma, Corey T McMillan, Tracy R Melzer, Bratislav Misic, Philip Mosley, Conor Owens-Walton, Laura M Parkes, Clelia Pellicano, Fabrizio Piras, Kathleen L Poston, Mario Rango, Christian Rummel, Petra Schwingenschuh, Melanie Suette, Paul M Thompson, Duygu Tosun, Chih-Chien Tsai, Tim D van Balkom, Odile A van den Heuvel, Ysbrand D van der Werf, Eva M van Heese, Chris Vriend, Jiun-Jie Wang, Roland Wiest, Clarissa L Yasuda, Alain Dagher

**Affiliations:** Department of Neurology and Neurosurgery, Montreal Neurological Institute, McGill University, Montreal, Canada H3A 2B4; Centre for Advanced Research in Sleep Medicine, Hôpital du Sacré-Cœur de Montréal, Montreal, Canada H4J 1C5; Centre for Advanced Research in Sleep Medicine, Hôpital du Sacré-Cœur de Montréal, Montreal, Canada H4J 1C5; Faculty of Health and Medicine, Lancaster University, Lancaster LA1 4AT, UK; Division of Neuroscience and Experimental Psychology, Faculty of Biology, Medicine and Health, The University of Manchester, Manchester M13 9PL, UK; Department of Neurology, Amsterdam UMC, Vrije Universiteit Amsterdam, Amsterdam 1081 HV, The Netherlands; Neurodegeneration, Amsterdam Neuroscience, Amsterdam 1081 HV, The Netherlands; Social, Genetic and Developmental Psychiatry Centre, Institute of Psychiatry, Psychology and Neuroscience, King's College London, London SE5 8AF, UK; Department of Neurology, University of Campinas–UNICAMP, Campinas 13083-888, Brazil; Neurodegeneration, Amsterdam Neuroscience, Amsterdam 1081 HV, The Netherlands; Department of Anatomy and Neurosciences, Amsterdam UMC, Vrije Universiteit Amsterdam, Amsterdam 1081 HV, The Netherlands; Te Kura Mahi ā-Hirikapo, School of Psychology, Speech and Hearing, University of Canterbury, Christchurch 8041, New Zealand; New Zealand Brain Research Institute, Christchurch 8011, New Zealand; Department of Neurology, Inselspital, University Hospital Bern, University of Bern, Bern CH-3010, Switzerland; Department of Neurology and Center of Expertise for Parkinson & Movement Disorders, Donders Institute for Brain, Cognition and Behaviour, Radboud University Medical Center, Nijmegen 6525 GC, The Netherlands; Centre for Cognitive Neuroimaging, Donders Institute for Brain, Cognition and Behaviour, Radboud University, Nijmegen 6525 EN, The Netherlands; Department of Radiology and Medical Imaging, University of Virginia, Charlottesville, VA 22903, USA; MoVeRe Group, GIGA-CRC In Vivo Imaging, University of Liège, Liège 4000, Belgium; Department of Neurology, CHU Liège, Liège 4000, Belgium; Department of Neurology and Center of Expertise for Parkinson & Movement Disorders, Donders Institute for Brain, Cognition and Behaviour, Radboud University Medical Center, Nijmegen 6525 GC, The Netherlands; Centre for Cognitive Neuroimaging, Donders Institute for Brain, Cognition and Behaviour, Radboud University, Nijmegen 6525 EN, The Netherlands; Division of Clinical Neurology, Department of Clinical Neurosciences, Oxford Parkinson's Disease Centre, Nuffield, University of Oxford, Oxford OX3 9DU, UK; Imaging Genetics Center, Mark and Mary Stevens Neuroimaging and Informatics Institute, Keck School of Medicine, University of Southern California, Marina del Rey, CA 90033, USA; Department of Neurology and Center of Expertise for Parkinson & Movement Disorders, Donders Institute for Brain, Cognition and Behaviour, Radboud University Medical Center, Nijmegen 6525 GC, The Netherlands; Centre for Cognitive Neuroimaging, Donders Institute for Brain, Cognition and Behaviour, Radboud University, Nijmegen 6525 EN, The Netherlands; Division of Clinical Neurology, Department of Clinical Neurosciences, Oxford Centre for Integrative Neuroimaging, Oxford Parkinson's Disease Centre, Nuffield, University of Oxford, Oxford OX3 9DU, UK; Neurodegeneration, Amsterdam Neuroscience, Amsterdam 1081 HV, The Netherlands; Department of Anatomy and Neurosciences, Amsterdam UMC, Vrije Universiteit Amsterdam, Amsterdam 1081 HV, The Netherlands; Department of Neurology, Perelman School of Medicine, University of Pennsylvania, Philadelphia, PA 19104, USA; Te Kura Mahi ā-Hirikapo, School of Psychology, Speech and Hearing, University of Canterbury, Christchurch 8041, New Zealand; New Zealand Brain Research Institute, Christchurch 8011, New Zealand; Department of Medicine, University of Otago, Christchurch 8011, New Zealand; Pacific Radiology Canterbury, Christchurch 8031, New Zealand; Department of Neurology and Neurosurgery, Montreal Neurological Institute, McGill University, Montreal, Canada H3A 2B4; QIMR Berghofer Medical Research Institute, Queensland 4006, Australia; Imaging Genetics Center, Mark and Mary Stevens Neuroimaging and Informatics Institute, Keck School of Medicine, University of Southern California, Marina del Rey, CA 90033, USA; Division of Psychology, Communication and Human Neuroscience, School of Health Sciences, Faculty of Biology, Medicine and Health, The University of Manchester, Manchester M13 9PL, UK; Geoffrey Jefferson Brain Research Centre, Manchester Academic Health Science Centre, Northern Care Alliance & University of Manchester, Manchester M6 8HD, UK; Laboratory of Neuropsychiatry, IRCCS Santa Lucia Foundation, Rome 00179, Italy; Laboratory of Neuropsychiatry, IRCCS Santa Lucia Foundation, Rome 00179, Italy; Department of Neurology & Neurological Sciences, Movement Disorders, Stanford University, Palo Alto, CA 94304, USA; Excellence Interdepartmental Center for Advanced MR Techniques and Department of Neurosciences, Neurology Unit, Parkinson's Disease Center, Fondazione Cà Granda, IRCCS, Ospedale Policlinico, University of Milan, Milan 20122, Italy; Support Center for Advanced Neuroimaging (SCAN), University Institute of Diagnostic and Interventional Neuroradiology, University Hospital Bern, Bern CH-3010, Switzerland; Department of Neurology, Medical University of Graz, Graz 8036, Austria; Department of Neurology, Medical University of Graz, Graz 8036, Austria; Imaging Genetics Center, Mark and Mary Stevens Neuroimaging and Informatics Institute, Keck School of Medicine, University of Southern California, Marina del Rey, CA 90033, USA; Department of Radiology and Biomedical Imaging, University of California SanFrancisco, San Francisco, CA 94143, USA; Healthy Ageing Research Center, Chang Gung University, Taoyuan City 33302, Taiwan; Neurodegeneration, Amsterdam Neuroscience, Amsterdam 1081 HV, The Netherlands; Department of Anatomy and Neurosciences, Amsterdam UMC, Vrije Universiteit Amsterdam, Amsterdam 1081 HV, The Netherlands; Department of Psychiatry, Amsterdam UMC, Vrije Universiteit Amsterdam, Amsterdam 1081 HV, The Netherlands; Neurodegeneration, Amsterdam Neuroscience, Amsterdam 1081 HV, The Netherlands; Department of Anatomy and Neurosciences, Amsterdam UMC, Vrije Universiteit Amsterdam, Amsterdam 1081 HV, The Netherlands; Department of Psychiatry, Amsterdam UMC, Vrije Universiteit Amsterdam, Amsterdam 1081 HV, The Netherlands; Neurodegeneration, Amsterdam Neuroscience, Amsterdam 1081 HV, The Netherlands; Department of Anatomy and Neurosciences, Amsterdam UMC, Vrije Universiteit Amsterdam, Amsterdam 1081 HV, The Netherlands; Neurodegeneration, Amsterdam Neuroscience, Amsterdam 1081 HV, The Netherlands; Department of Anatomy and Neurosciences, Amsterdam UMC, Vrije Universiteit Amsterdam, Amsterdam 1081 HV, The Netherlands; Department of Anatomy and Neurosciences, Amsterdam UMC, Vrije Universiteit Amsterdam, Amsterdam 1081 HV, The Netherlands; Department of Psychiatry, Amsterdam UMC, Vrije Universiteit Amsterdam, Amsterdam 1081 HV, The Netherlands; Brain Imaging, Amsterdam Neuroscience, Amsterdam 1081 HV, The Netherlands; Department of Medical Imaging and Radiological Sciences, Chang Gung University, Taoyuan City 33302, Taiwan; Support Center for Advanced Neuroimaging (SCAN), University Institute of Diagnostic and Interventional Neuroradiology, University Hospital Bern, Bern CH-3010, Switzerland; Department of Neurology, University of Campinas–UNICAMP, Campinas 13083-888, Brazil; Brazilian Institute of Neuroscience and Neurotechnology, Campinas 13083-888, Brazil; Department of Neurology and Neurosurgery, Montreal Neurological Institute, McGill University, Montreal, Canada H3A 2B4

**Keywords:** Parkinson’s disease, neurodegeneration, structural MRI, connectivity, imaging transcriptomics

## Abstract

Parkinson’s disease is associated with extensive structural brain changes. Recent work has proposed that the spatial pattern of disease pathology is shaped by both network spread and local vulnerability. However, few studies have assessed these biological frameworks in large patient samples across disease stages.

Analysing the largest imaging cohort in Parkinson’s disease to date (*n* = 3096 patients), we investigated the roles of network architecture and local brain features by relating regional abnormality maps to normative profiles of connectivity, intrinsic networks, cytoarchitectonics, neurotransmitter receptor densities and gene expression.

We found widespread cortical and subcortical atrophy in Parkinson’s disease to be associated with advancing disease stage, longer time since diagnosis and poorer global cognition. Structural brain connectivity best explained cortical atrophy patterns in Parkinson’s disease and across disease stages. These patterns were robust among individual patients. The precuneus, lateral temporal cortex and amygdala were identified as likely network-based epicentres, with high convergence across disease stages. Individual epicentres varied significantly among patients, yet they consistently localized to the default mode and limbic networks. Furthermore, we showed that regional overexpression of genes implicated in synaptic structure and signalling conferred increased susceptibility to brain atrophy in Parkinson’s disease.

In summary, this study demonstrates in a well-powered sample that structural brain abnormalities in Parkinson’s disease across disease stages and within individual patients are influenced by both network spread and local vulnerability.

## Teaser

Vo *et al.* analysed brain imaging data from over 3000 people with Parkinson’s disease and found widespread structural brain changes, including in early stages. These patterns were shaped by both brain network connectivity and local biological features such as gene expression and receptor density.

## Introduction

Parkinson’s disease (PD) is a progressive neurodegenerative disorder marked by extensive structural changes in the brain, affecting both cortical and subcortical regions.^1-3^ However, the spatial pattern of atrophy is not uniform across the brain. Some regions show greater vulnerability to disease pathology than others. This raises questions as to what the underlying factors are that shape and drive the spread of pathology in PD.

Early post-mortem studies describe a distribution pattern of Lewy pathology in PD that appears to map onto large-scale intrinsic networks in the brain.^4,5^ This Braak staging implies that the spread of PD pathology is not random, but constrained by the organization of the underlying connectome.^6,7^ It has been hypothesized that this network spreading process involves the propagation of misfolded alpha-synuclein protein via neuronal synapses in a prion-like manner.^7,8^ The primary support for this hypothesis comes from animal studies that traced the neuronal spread of injected alpha-synuclein.^9-11^ In addition, there is mounting evidence in support of this hypothesis derived from patient populations using non-invasive brain imaging and computational modelling.^1,12-17^ However, the progression of PD pathology does not always align neatly with the Braak staging framework,^18^ suggesting that network spreading is not the only driver of pathology. Indeed, local vulnerability features, such as cellular composition,^19^ metabolic demands^20^ or gene expression,^21^ may predispose certain brain regions to disease pathology and damage. Studies employing imaging transcriptomics to explore the relationship between brain morphometry in PD and transcriptional gene activity, for example, have shown that the local expression of genes related to synaptic, mitochondrial and metabolic activity render certain brain regions particularly susceptible to atrophy.^12,13,22,23^

Studies of structural brain abnormalities, as well as network spread and local vulnerability in PD, have so far been limited to relatively small samples of clinically heterogeneous patients. Moreover, these studies are complicated by variability in analytic approaches between different study sites. The Enhancing Neuroimaging Genetics through Meta-analysis PD (ENIGMA-PD) working group is an international collaboration across multiple centres that has curated and harmonized the largest imaging dataset in PD to date.^3,24,25^ Here, we analysed this well-powered dataset to map cortical and subcortical atrophy in PD, across disease stages and within single subjects. We then related these spatial atrophy patterns to normative network models. Next, we investigated whether PD atrophy mapped onto specific macro-scale brain systems. Finally, we related atrophy patterns to local gene expression profiles and explored their biological relevance. We hypothesized that brain atrophy in PD is shaped by both the connectivity between regions, which may facilitate the spread of pathology, and the local biological factors that confer vulnerability to disease within regions, with each explaining unique variance in the spatial atrophy pattern.

## Materials and methods

### Participants

The ENIGMA-PD working group aggregated 3D volumetric T1-weighted brain MRI and clinical data from PD patients and healthy controls (HCs) across 23 international contributing sites (Supplementary Table 1). Brain imaging was available from 3216 PD patients and 1480 HCs. Individual site MRI scanning protocols and participant inclusion/exclusion criteria are detailed in Supplementary Table 2. Clinical information from PD patients included Hoehn and Yahr (HY) stage score,^26^ time since diagnosis (in years) and Montreal Cognitive Assessment (MoCA) score.^27^ HY scores ranged from 1 (i.e. unilateral motor impairment) to 5 (i.e. confinement to bed or wheelchair). We used a modified HY classification such that intermediate scores HY 1.5 and 2.5 were regrouped into HY 2, and HY 4 and 5 were combined due to smaller group sizes. All participants provided written informed consent to their local site before participating in site-specific studies, which were approved by the respective local ethics committee and institutional review board. Anonymized imaging and clinical data were shared with the ENIGMA-PD working group.

### Structural brain morphometry in PD

Each contributing site collected and processed MRI data using a standardized FreeSurfer 5.3 pipeline,^28^ extracting 68 regional cortical thickness, 68 cortical surface area, 16 subcortical volume, and total intracranial volume estimates according to the Desikan-Killiany atlas.^29^ These regional brain estimates were visually inspected for quality following standard ENIGMA protocols (http://enigma.usc.edu/protocols/imaging-protocols) and shared with the ENIGMA-PD investigators. Participants younger than 40 years of age or those with more than 50% missing data after quality control were excluded from the analysis. This resulted in a final sample of 3096 PD patients and 1262 HCs.

The 23 sites contributed a combined 53 cohorts, each with different cohort-specific scanning and clinical testing environments. Accordingly, we first harmonized brain estimates using ComBat, a Bayesian statistical harmonization method designed to account for batch effects in multi-site MRI studies, correcting for age and sex covariates.^30^ A small subset of cohorts (9 of 53 cohorts) had data available only from PD patients but no matched HCs. To rule out that our results may have been influenced by inclusion of these unmatched cohorts during data harmonization, we conducted a robustness test by comparing group average brain maps derived from data harmonized before versus after excluding PD-only cohorts from the sample. We found comparable abnormality patterns between these two samples for all measures (Supplementary Fig. 1A and C). To validate that our harmonization procedure successfully removed cohort effects, we ran linear models including cohort as a covariate on both the unadjusted and ComBat-adjusted data and compared the partial *R*^2^ of cohort between these models. Across all regional brain estimates, we consistently found that the variance explained by cohort differences was successfully removed by ComBat (Supplementary Tables 3–5). Any remaining missing values were imputed based on the mean of the group (i.e. PD or HC) and disease stage (i.e. HY score) to which a given participant belonged.

For each brain measure estimate, we generated *w*-score maps for each PD patient.^31^ This procedure is analogous to *z*-scoring with the additional adjustment for age and sex covariates. Since cortical surface area and subcortical volumes scale with head size,^32^ total intracranial volume was also included as a covariate in their *w*-score models. Given the PD and HC groups were significantly different in terms of age and sex and the potential for this to influence *w*-score estimates, we ran a sensitivity analysis comparing group average brain maps derived from data with versus without HY stage stratification, propensity score matching with replacement using the MatchIt tool (Supplementary Fig. 1A and C).^33^ We found comparable abnormality patterns between these two samples for all measures.

For the *w*-scoring procedure, linear regressions between regional brain estimates and age and sex were first performed in the HC reference cohort. Then, regional *w*-scores were calculated for each PD patient using the following formula:


(1) $$w_{i}=\;\frac{raw_{i}-expected_{i}}{SD_{residual}}$$

where $w_{i}$ is the *w*-score of region *i*, $raw_{i}$ is the estimate value at region *i* observed in the patient, $expected_{i}$ is the estimate value at region *i* expected in the HC group given the patient’s age and sex (estimate∼age+sex), and $SD_{residual}$ is the standard deviation of the residuals in HCs. Here, negative *w*-scores reflected lower estimates (i.e. atrophy), whereas positive *w*-scores indicated higher values in PD than expected in HCs. Group average maps of PD-related deviations for each estimate were generated from the mean regional *w*-scores across all PD patients. One-sample *t*-tests examined if group average *w*-scores in PD significantly deviated from HCs (i.e. test if average PD had a *w*-score that differed from 0). Results were corrected for multiple comparisons using the false discovery rate (FDR) method.^34^ Five-fold cross-validation with permutation testing (*n* = 1000) demonstrated that the patterns of brain abnormality in PD were robust and consistent across different subsets of the sample (Supplementary Table 6 and Supplementary Fig. 2A and B).

### Relationship between structural brain abnormalities and clinical measures

We tested whether maps of PD-related brain abnormalities were associated with clinical measures, controlling for age and sex. Clinical measures included HY stage, time since diagnosis (in years), and global cognition as estimated by MoCA scores (i.e. lower scores reflect poorer cognition).^27^ Given that HY scores are ordinal in nature, we used non-parametric, rank-based partial correlations that model the monotonic relationship between variables of interest. For the other continuous variables, we used linear regression models. To ensure consistent interpretation of correlations, we inverted MoCA scores to align with the directionality of the other clinical measures so that higher values reflected greater disease severity. Results were FDR-corrected for multiple comparisons separately for each brain and clinical measure.^34^

### Spatial null models

The inherent autocorrelation among brain regions can artificially inflate correlations when testing the spatial overlap between two brain maps.^35^ To mitigate this issue, we evaluated the statistical significance of such correlations using spatial null models, or ‘spin tests’,^36,37^ whenever appropriate. Null models were generated using the *netneurotools* toolbox (https://netneurotools.readthedocs.io/en/latest/). Coordinates of cortical surface parcels were first projected onto the surface of spheres, then randomly rotated for one hemisphere and mirrored to the other. Subsequently, cortical surface data were reassigned with the values of the closest rotated parcel. This procedure was repeated 1000 times to construct a null distribution that preserved the spatial autocorrelation of the original surface map and provided a benchmark against which we could compare the original observation. For subcortical data, instead of spherical projection, parcels were randomly shuffled to generate permuted null models.^38^

### Normative connectivity network models

Structural and functional connectivity networks were obtained from the *enigmatoolbox*,^38^ derived from diffusion-weighted imaging and resting-state functional MRI data in a cohort of unrelated healthy adults from the Human Connectome Project.^39^ Details on how these data were preprocessed to construct connectivity networks are provided in the Supplementary material.

Structural connectivity networks were built from preprocessed diffusion-weighted imaging data using MRtrix3.^40^ This involved performing tractography constrained to anatomically derived tissue types (i.e. cortical and subcortical grey matter, white matter and CSF),^41^ estimation of multi-shell and multi-tissue response functions,^42^ and constrained spherical deconvolution and intensity normalization.^43^ The initial tractogram was generated with 40 million streamlines (max length = 250, fractional anisotropy cut-off = 0.06). Spherical deconvolution-informed filtering of tractograms with SIFT2^44^ was used to reconstruct whole brain streamlines weighted by cross-sectional multipliers. Individual structural connectomes were generated by mapping the reconstructed streamlines onto 68 cortical and 14 subcortical regions (the lateral ventricles were excluded). A distance-dependent thresholding procedure,^45^ which preserves the edge length distribution of individual connectomes, and log transformation were used to define a group-average structural connectivity network in which each connection represents the number of streamlines or fibre density between two brain regions. Functional connectivity networks were constructed by performing pairwise correlations between the time series of 68 cortical and 14 subcortical regions. Negative connections were set to zero. Individual functional connectomes were *z*-transformed and averaged across subjects to generate a group-average functional connectivity network.

Four normative network models were used in the present study: (i) cortico-cortical structural network; (ii) subcortico-cortical structural network; (iii) cortico-cortical functional network; and (iv) subcortico-cortical functional network. Cortico-cortical networks were based on connections among 68 cortical regions (68 × 68 matrix). Subcortico-cortical networks featured connections between 14 subcortical and 68 cortical regions (14 × 68 matrix).

### Network-based disease exposure

**Figure 1:**
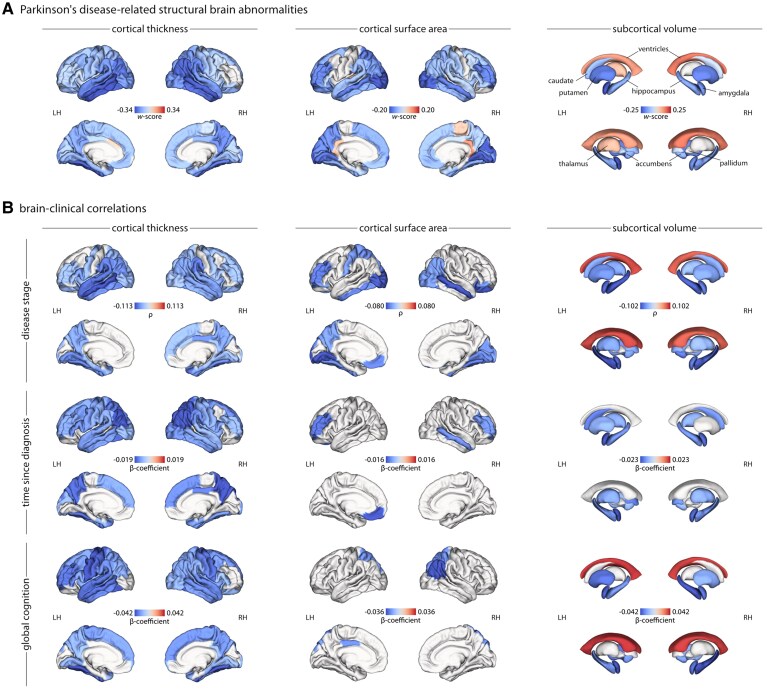
**Structural brain abnormalities in Parkinson’s disease and their clinical correlates**. (**A**) *W*-score maps of cortical thickness, cortical surface area, and subcortical volume deviations reveal a widespread pattern of atrophy in Parkinson’s disease (PD). More negative *w*-scores (or darker blue regions) represent lower estimates or greater atrophy in PD patients relative to what would be expected in the healthy reference group. (**B**) Partial rho (*ρ*) and *β*-coefficient maps of brain-clinical correlations between cortical thickness, cortical surface area, and subcortical volume deviations and Hoehn and Yahr (HY) disease stage, time since diagnosis (in years) and global cognition (Montreal Cognitive Assessment scores), controlling for age and sex effects. Overall, more negative deviations in brain measures were related to advancing disease stage, longer disease durations and poorer cognition. For display purposes, only regions surviving false discovery rate (FDR) correction for multiple comparisons (*P*_FDR_ < 0.05) are shown. LH = left hemisphere; RH = right hemisphere.

We examined how the patterns of PD-related atrophy are shaped by structural and functional connectivity across PD disease stages. Specifically, we tested if the atrophy measured at a given region (or node) was related to atrophy across its structurally and functionally connected neighbours.^46,47^ Nodal atrophy refers to the regional *w*-scores from the cortical thickness and subcortical volume maps, which showed the most sensitivity to PD-related atrophy and clinical features (Fig. 1A and B). Neighbourhood atrophy of a given node was defined as the average *w*-score across its structurally connected neighbour regions weighted by the strength of connectivity, described in the following formula:


(2) $$A_{i}=\frac{1}{N_{i}}\overset{N_{i}}{\underset{\;j\neq i,\;j=1}{\sum }}\;a_{j}\cdot conn_{ij}$$

where $A_{i}$ is the neighbourhood atrophy of node *i*, $a_{j}$ is the atrophy of the *j*-th neighbour of node *i*, $conn_{ij}$ is the strength of the connection between nodes *i* and *j*, and $N_{i}$ is the total number of structurally connected neighbours to node *i* (i.e. node degree). Note that neighbourhood atrophy is normalized by the node degree ($N_{i}$) and is thus made independent of nodal atrophy. Self-connections between a node and itself are also excluded (j≠i).

For each network model, we tested the relationship between node and neighbourhood atrophy using Spearman rank correlations. Each test was compared against spatial null models to determine statistical significance. Finally, we repeated this analysis separately on the group average, disease stage, and single-subject atrophy maps.

### Network-based epicentre likelihoods

**Figure 2:**
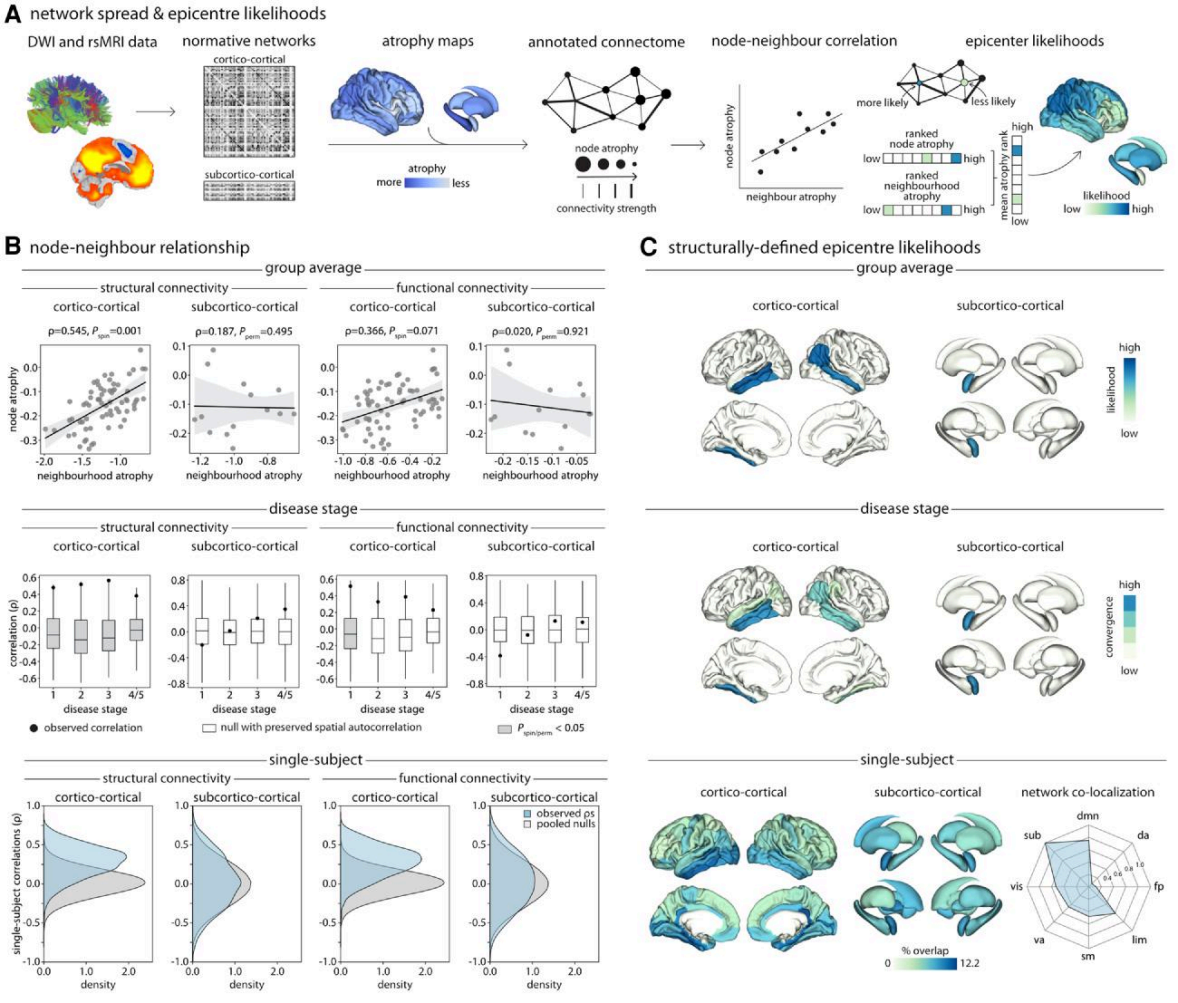
**Network architecture shapes the pattern of atrophy in Parkinson’s disease**. (**A**) Schematic of network-based disease exposure and epicentre likelihood workflows. Structural and functional connectivity was defined by cortico-cortical and subcortico-cortical normative network models derived from an unrelated cohort of healthy participants. This information was used to relate regional abnormality (or ‘node atrophy’) to the average abnormality across connected neighbour regions (or ‘neighbourhood atrophy’). The mean rank of node and neighbourhood atrophy was used to identify regions as likely epicentres. (**B**) Node-neighbourhood atrophy correlations revealed that atrophy patterns were best explained by cortico-cortical structural network models (*top row*), across disease stages (*middle row*) and in a large proportion of individual patients (*bottom row*), followed by cortico-cortical functional network models. Neither subcortico-cortical structural or functional network models explained node-neighbourhood coupling, however. (**C**) Epicentre likelihoods identified the precuneus, lateral temporal cortex, and amygdala as network-based epicentres (*top row*), which were consistently identified across disease stages (*middle row*). Note that single-subject epicentres did not frequently generalize across individual patients (*bottom row*), with a peak overlap of only 12.2% of the entire sample. However, these epicentres were found to co-localize within common networks, namely the default mode and limbic networks as well as the subcortex, across a large proportion of patients. For all brain visualizations, only regions surviving spatial null testing (*P*_spin/perm_ < 0.05) are displayed. da = dorsal attention; dmn = default mode; DWI = diffusion weighted imaging; fp = frontoparietal; lim = limbic; rsMRI = resting state MRI; sm = sensorimotor; sub = subcortex; va = ventral attention; vis = visual.

For each network model, we ranked brain regions based on their nodal atrophy and neighbourhood atrophy (Fig. 2A). The epicentre likelihood of a given region was defined as the average of node and neighbourhood atrophy ranks, so that those regions with greater atrophy that are also connected to neighbourhoods with greater atrophy are considered more likely epicentres.^46,47^ These likelihoods were tested against spatial null models for statistical significance. It is important to note that a network-based epicentre does not necessarily implicate a region as the initiation site of disease. Instead, the term describes a region with an atrophy and connectivity profile ideal for a propagator of disease pathology.

Epicentre likelihoods were examined separately for the group average, disease stage, and single-subject atrophy maps. For disease stage atrophy maps, after identifying regions with significant epicentre likelihoods for each HY stage, we explored the convergence of these epicentre regions across the four disease stages. For the single-subject atrophy maps, we similarly looked at the convergence of significant epicentre regions across individual PD patients. Anticipating a large degree of heterogeneity between individualized epicentre maps, epicentre regions were also mapped to intrinsic brain networks^48^ to test if they might co-localize to common circuitry.

### Spatial overlap between atrophy and brain annotations

We investigated the spatial correspondence between PD-related atrophy and annotations of discrete brain systems, including intrinsic networks^48^ and cytoarchitectonic tissue classes.^49,50^ The Yeo intrinsic networks^48^ classify cortical regions into seven distinct resting-state networks: default mode, frontoparietal, limbic, ventral attention, dorsal attention, sensorimotor and visual networks. Similarly, the von Economo classification^49,50^ assigns cortical regions into seven cytoarchitectonic types: insular, limbic, primary sensory, primary/secondary sensory, association 1 and 2, and primary motor classes. For cortical thickness and surface area maps, we computed the mean *w*-score within each network or tissue class to localize the macroscale distribution of atrophy in PD. Observed mean atrophy within each system was compared against null mean distributions derived from spatial null models.

We also explored the spatial overlap between PD-related atrophy and 18 receptor and transporter densities that span nine neurotransmitter systems.^51,52^ Volumetric radiotracer maps from a total cohort of over 1200 healthy individuals were obtained from https://github.com/netneurolab/hansen_receptors. This included data for acetylcholine (α_4_β_2_, M_1_, VAChT), cannabinoid (CB_1_), dopamine (D_1_, D_2_, DAT), GABA (GABA_A_/B_Z_), histamine (H_3_), glutamate (mGluR_5_, NMDA), norepinephrine (NET), opioid (MOR) and serotonin (5-HT_1A_, 5-HT_1B_, 5-HT_2A_, 5-HT_4_, 5-HT_6_, 5-HTT). These tracer maps were parcellated according to the Desikan-Killiany atlas and individually *z*-scored using the *neuromaps* toolbox.^52^ For tracers with multiple available maps, we combined them into a single map using weighted averaging. Spearman rank correlations assessed the relationship between each cortical thickness, cortical surface area, and subcortical volume map and each neurotransmitter map. Correlations were tested against spatial null models and FDR-corrected for multiple comparisons.

### Gene expression profiles

Gene expression data were obtained from the Allen Human Brain Atlas^53^ and processed with the *abagen* toolbox.^54^ The dataset is composed of microarray data derived from six post-mortem brains (five males, one female; mean age ± standard deviation = 42.5 ± 13.4 years). Probes were reannotated following previous recommendations.^55^ Those with a signal-to-noise ratio greater than 50% were retained. If there were multiple probes of the same gene, the one with the most consistent pattern of regional expression across donors was selected. This procedure resulted in a total of 15 633 genes retained. Samples were assigned to parcels of the Desikan-Killiany atlas.^29^ This sample-to-parcel matching was restricted to each hemisphere and within gross structural divisions to minimize assignment errors. When a probe was not found directly within a parcel, the nearest sample up to 2 mm away was selected. If no probes were found within 2 mm of a parcel, then the sample closest to the centroid of the parcel across all donors was chosen. Samples unable to be assigned to a parcel were discarded. Expression values were normalized across genes using a scaled robust sigmoid function and rescaled to the unit interval, then normalized across tissue samples using the same procedure. Regional gene expression profiles were obtained by first averaging across probes belonging to the same parcels separately for each donor, then averaging across donors. Given that data from the right hemisphere were available from only two of the six donors, the current analysis was restricted to the left hemisphere. In addition, due to the large transcriptional difference between the cortex and subcortex^56^ and relatively few subcortical regions to assess (i.e. seven parcels), we restricted our analysis to the cortex. Regional gene expression profiles were averaged across donors to construct a 34 region × 15 633 gene expression matrix that was used for partial least squares analysis.

### Partial least squares analysis

We used partial least squares (PLS) analysis^57,58^ to identify the patterns of gene expression associated with PD-related atrophy. This approach identifies latent variables that explain the maximum covariance between matrices *X* (gene expression: 34 regions × 15 633 genes) and *Y* (atrophy: 34 regions). The statistical significance of latent variables was assessed against the variance observed in 1000 spatial null models. For each significant latent variable identified, the contribution of individual genes was determined through bootstrap resampling. This procedure involves shuffling matrices *X* and *Y*, then repeating the PLS analysis 1000 times to generate a null distribution, and using the standard errors to estimate the weight (or contribution) of each gene. Bootstrap ratios, which are interpreted in the same way as *z*-scores, were calculated as the ratio of each gene weight to its bootstrap-estimated standard error. Genes with larger bootstrap ratios contribute more significantly and reliably to a given latent variable. Ranked gene lists based on these bootstrap ratios were submitted to gene set enrichment analysis (GSEA).^59^

### Gene set enrichment analysis

To investigate the biological relevance of gene expression correlates of PD-related atrophy, we performed GSEA using the WebGestalt platform (https://www.webgestalt.org) and the Gene Ontology knowledge base (https://geneontology.org). GSEA tests whether the most positively and negatively weighted genes in a ranked gene list, derived here from bootstrap resampling, occur more frequently than expected by random chance and identifies the biological process and cellular component terms associated with these significant genes. The minimum and maximum number of genes for enrichment was set to 3 and 2000, respectively. Results were adjusted by running 1000 random permutations, followed by FDR correction for multiple comparisons. We report and interpret the 10 most positively and negatively weighted terms.

## Results

### Participants

Table 1 displays the demographic and clinical details of the participants included in the study. Supplementary Table 1 details the demographic and clinical characteristics of the participants for each contributing site, and Supplementary Table 2 details the inclusion/exclusion criteria. The PD group was significantly older than the HC group [*t*(2522) = −5.24, *P* < 0.001]. Although the age range of the HC group (40–85 years) did not entirely cover the span of the PD group (40–89 years), only seven PD patients (<1%) exceeded this range, which suggests an overall sufficient overlap for deriving atrophy maps using *w*-scoring. Critically, a Levene’s test demonstrated that the two groups did not differ in the variance of ages [*W*(1,4358) = 1.00, *P* = 0.316], indicating comparable age distributions. The proportion of males to females was also significantly different between groups (*χ*^2^ = 44.95, *P* < 0.001). Despite these group differences in age and sex, a sensitivity analysis comparing atrophy maps derived from the complete sample and an age- and sex-matched subsample (Supplementary Table 7) demonstrated comparable patterns of structural abnormalities (Supplementary Fig. 1A and B). For PD patients with available information on HY disease stages^26^ (82.5% of total sample), the majority were classified as HY stage 2.

**Table 1:** ENIGMA-PD sample demographics and clinical details

Group	HY stage	*n*	Age (mean ± SD)	Sex (% female)	Time since diagnosis (mean ± SD)	MoCA (mean ± SD)
HC	–	1262	62.09 ± 9.25	47.31	–	27.88 ± 1.70^a^
PD	–	3096	63.69 ± 9.09	36.30	5.18 ± 5.11^b^	25.89 ± 3.29^a^
	1	466	60.15 ± 8.63	40.56	2.38 ± 2.52	27.20 ± 2.32
	2	1596	63.90 ± 8.80	34.21	4.50 ± 4.43	26.26 ± 2.91
	3	397	66.05 ± 9.43	40.81	7.76 ± 5.98	24.80 ± 3.74
	4/5	96	67.48 ± 9.38	38.54	13.05 ± 6.42	21.41 ± 4.88

HC = healthy controls; HY stage = Hoehn and Yahr disease stage; MoCA = Montreal Cognitive Assessment score; PD = Parkinson’s disease; SD = standard deviation.

^a^Information available in 1796 of 3096 patients and 454 of 1262 controls.

^b^Information available in 2879 of 3096 patients.

### Widespread structural brain abnormalities in Parkinson’s disease

To investigate PD-related deviations in age- and sex-adjusted *w*-maps, one-sample *t*-tests compared regional mean *w*-scores to zero. We found statistically significant and diffuse negative deviations in cortical thickness that were most pronounced in parietal and temporal regions (Fig. 1A and Supplementary Table 8). Cortical surface area was also generally lower in PD, with the greatest negative deviations in occipital and middle frontal cortex (Fig. 1A and Supplementary Table 9). Finally, grey matter volumes were lower across the majority of subcortical nuclei, with peak negative deviations in the putamen and amygdala. The volume of the left thalamus and lateral ventricles was higher than expected, however (Fig. 1A and Supplementary Table 10). When stratified by HY disease stage scores, the number of regions demonstrating significant deviations and the magnitude of these abnormalities increased with higher disease stage (Supplementary Fig. 3).

Next, we explored the relationships between regional brain abnormalities and clinical scores in PD, adjusting for age and sex. Rank-based partial correlations revealed regional deviations became more negative with higher disease stages for all brain measures, except for ventricular volumes that displayed the inverse relationship. These patterns are consistent with a progressive atrophic process in PD. Similarly, linear regressions showed time since diagnosis was negatively correlated with each brain measure, such that longer durations corresponded with more negative deviations in regional cortical thickness, cortical surface area, and subcortical volume (Fig. 1B and Supplementary Tables 11–13). Global cognition, assessed with the MoCA,^27^ was also negatively correlated with each brain measure such that poorer cognition was related to more negative deviations in cortical thickness and subcortical grey matter volume but more positive deviations in the lateral ventricles.

### Network architecture shapes cortical atrophy across disease stages and in individual patients

To test the hypothesis that the atrophy pattern in PD is shaped by network architecture, we related regional abnormality (or ‘nodal atrophy’) to the abnormality across structurally connected regions (or ‘neighbourhood atrophy’) weighted by the strength of connectivity (Fig. 2A). In the group average maps (Fig. 2B, top row, and Supplementary Table 14), nodal atrophy was positively correlated with neighbourhood atrophy for both structural (*ρ* = 0.545, *P*_spin_ = 0.001) and functional (*ρ* = 0.366, *P*_spin_ = 0.071) cortico-cortical network models, although the latter was no longer statistically significant after spatial null testing. However, structural and functional subcortico-cortical network models poorly explained the node-neighbourhood correlation (both *P*_perm_ > 0.05). For the disease stage maps (Fig. 2B, middle row, and Supplementary Table 15) in which patients were stratified by their HY stage, we found that the atrophy pattern was again best explained by cortico-cortical network models. Structural connectivity accounted for cortical atrophy patterns across HY stages 1 to 4/5, and this finding was robust when tested against spatial null models. Similarly, functional connectivity informed the cortical atrophy pattern across early HY stages (HY 1–3), but node-neighbourhood atrophy did not survive spatial null testing beyond HY stage 1 (Fig. 2B, middle row, and Supplementary Table 15). In subcortico-cortical network models, subcortical atrophy patterns were not well explained by either structural or functional connectivity at any disease stage (all *P*_perm_ > 0.05).

Finally, for single-subject atrophy maps (Fig. 2B, bottom row), we observed high stability in node-neighbourhood atrophy coupling for both structural (*P*_spin_ < 0.05 in 59.43% of patients) and functional (*P*_spin_ < 0.05 in 59.04% of patients) cortico-cortical network models. In contrast, there was low stability between node-neighbourhood atrophy in structural (*P*_perm_ < 0.05 in 5.98% of patients) and functional (*P*_perm_ < 0.05 in 6.40% of patients) subcortico-cortical models. Despite the large degree of heterogeneity expected across these individualized atrophy maps, a large proportion of PD patients still demonstrated connectivity-based cortical atrophy patterns observed in the group average.

### Network-based epicentres converge across disease stage and co-localize to common networks

In the group average maps (Fig. 2C, top row), we identified the right precuneus and bilateral lateral temporal cortex, and left amygdala as having high epicentre likelihoods using both cortico-cortical and subcortico-cortical network models, respectively. These likely epicentres demonstrated a high degree of atrophy and were themselves connected to neighbourhoods of highly atrophied regions. Similarly, these regions were consistently identified as likely epicentres across the disease stage maps (Fig. 2C, middle row), showing high convergence across the four HY stages. Network models defined by structural and functional connectivity resulted in the same epicentres (Supplementary Fig. 4A and B).

Repeating this approach in single-subject atrophy maps (Fig. 2C, bottom row), the right isthmus cingulate showed the highest percentage of convergence across individual PD patients—although the maximum overlap was only 12.2% of patients. However, when we mapped each PD patient’s likely epicentres to intrinsic brain networks, these disparate individualized epicentres co-localized to the default mode and limbic networks (75.48% and 60.92% of PD patients, respectively) and the subcortex (100% of PD patients).

### Cortical abnormalities are distributed in specific brain systems

**Figure 3:**
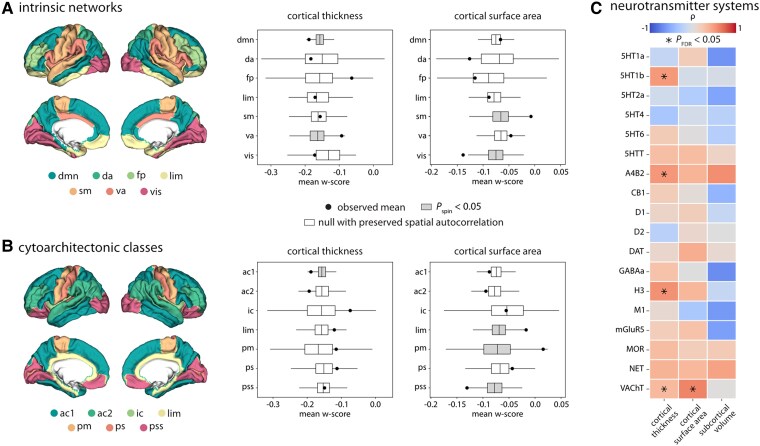
**Distribution of Parkinson’s disease-related atrophy in specific brain systems**. Cortical thickness (*left*) and cortical surface area (*right*) abnormalities in Parkinson’s disease (PD) are localized within (**A**) intrinsic resting networks^48^ and (**B**) cytoarchitectonic tissue classes.^49,50^ (**C**) Correlations between regional brain atrophy and expression of 18 neurotransmitter systems: acetylcholine (α_4_β_2_, M_1_, VAChT), cannabinoid (CB_1_), dopamine (D_1_, D_2_, DAT), GABA (GABA_A/BZ_), histamine (H_3_), glutamate (mGluR_5_), norepinephrine (NET), opioid (MOR) and serotonin (5-HT1_A_, 5-HT1_B_, 5-HT2_A_, 5-HT_4_, 5-HT_6_, 5-HTT). *Correlations that survived both spatial null testing (*P*_spin/perm_ < 0.05) and false discovery rate (FDR) correction for multiple comparisons (*P*_FDR_ < 0.05). ac1/2 = association 1/2; dmn = default mode; da = dorsal attention; fp = frontoparietal; ic = insular; lim = limbic; pm = primary motor; ps = primary sensory; pss = primary/secondary sensory; sm = sensorimotor; va = ventral attention; vis = visual.

Beyond network structure, we also studied whether local brain features contributed to the atrophy pattern in PD. For functional resting state networks,^48^ mean cortical thickness deviations were more negative in the default mode network (*P*_spin_ = 0.049) but less so in the ventral attention network (*P*_spin_ = 0.015; Fig. 3A). For cytoarchitectonic classes,^49,50^ these deviations were also found to be more negative in association cortex (*P*_spin_ = 0.009; Fig. 3B). Mean cortical surface area deviations were mainly distributed in visual and sensorimotor networks, with more negative deviations in the visual (*P*_spin_ = 0.002) but less pronounced in the sensorimotor network (*P*_spin_ = 0.006; Fig. 3A). They were also relatively more negative in primary/secondary sensory cortex (*P*_spin_ = 0.006) but less severe in limbic (*P*_spin_ = 0.005) and primary motor systems (*P*_spin_ = 0.017; Fig. 3B).

We then asked if the pattern of brain abnormalities in PD was related to molecular profiles of neurotransmitter systems. Cortical thickness deviations were significantly related to several neurotransmitter distributions (Fig. 3C and Supplementary Table 16), including 5HT_1b_ receptor (*ρ* = 0.493, *P*_FDR_ = 0.018), nicotinic A_4_B_2_ receptor (*ρ* = 0.503, *P*_FDR_ = 0.024), histamine H_3_ receptor (*ρ* = 0.537, *P*_FDR_ = 0.018), and vesicular acetylcholine transporter (VAChT; *ρ* = 0.322, *P*_FDR_ = 0.031). Cortical surface area deviations were also significantly correlated with VAChT (*ρ* = 0.595, *P*_FDR_ = 0.018; Fig. 3C and Supplementary Table 17). In each case, there was a positive association between the brain measure and neurotransmitter receptor density, suggesting that greater atrophy was observed in regions with lower expression of these systems (Supplementary Fig. 5). No correlations involving subcortical volumes survived FDR correction for multiple comparisons, however (Supplementary Table 18).

### Cortical atrophy is associated with synapse-related gene expression profiles

**Figure 4:**
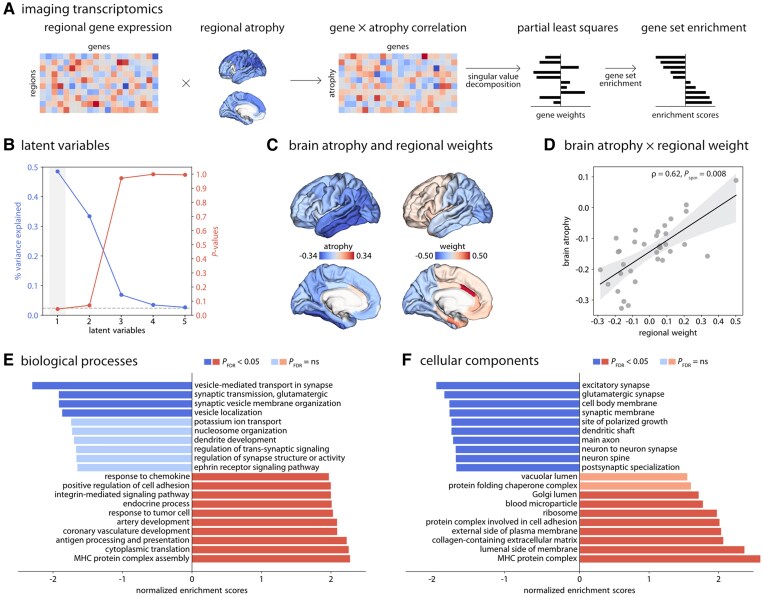
**Relationship between cortical atrophy in Parkinson’s disease and gene expression.** (**A**) Schematic of the imaging transcriptomics workflow. (**B**) Partial least squares (PLS) analysis identified a single latent variable (PLS1) that significantly explained 48.53% of the covariance between cortical thickness deviations and gene expression profiles. (**C**) Regional weights were (**D**) negatively correlated with regional brain atrophy, indicating that more positive weights were associated with greater cortical atrophy. Gene set enrichment analysis revealed that genes most correlated with cortical atrophy are enriched for (**E**) synaptic regulation and signalling and (**F**) synapse and neuron components. FDR = false discovery rate; ns = not significant.

To understand the biological and cellular underpinnings of the atrophy pattern in PD, we integrated our imaging findings with brain-wide gene expression data and explored the biological relevance of the associated genes (Fig. 4A). PLS analysis identified a single significant latent variable (PLS1) that explained 48.53% (*P*_spin_ = 0.044) of the covariance between the cortical atrophy pattern and gene expression (Fig. 4B). Regional weights associated with PLS1 were positively correlated with the cortical atrophy pattern (*ρ* = 0.620, *P*_spin_ = 0.008), such that more negatively weighted genes were associated with more negative deviations in cortical thickness (Fig. 4C and D).

Next, the biological relevance of identified genes was explored using GSEA. This analysis revealed that regions with greater cortical atrophy were found to overexpress genes related to synaptic signalling and regulation. Concerning biological processes (Fig. 4E and Supplementary Table 19), the genes most associated with cortical atrophy were enriched for terms such as ‘vesicle-mediated transport in synapse’ [normalized enrichment score (NES) = −2.305, *P*_FDR_ < 0.001], ‘synaptic transmission, glutamatergic’ (NES = −1.921, *P*_FDR_ = 0.045), ‘synaptic vesicle membrane organization’ (NES = −1.921, *P*_FDR_ = 0.031) and ‘vesicle localization’ (NES = −1.874, *P*_FDR_ = 0.044). Positively weighted genes were significantly enriched for diverse functional biological processes. In terms of cellular components (Fig. 4F and Supplementary Table 20), genes most strongly related to cortical atrophy were enriched for components localized to the synapse and neuron, such as ‘excitatory synapse’ (NES = −2.068, *P*_FDR_ = 0.009), ‘synaptic membrane’ (NES = −1.877, *P*_FDR_ = 0.014) and ‘main axon’ (NES = −1.826, *P*_FDR_ = 0.013). For genes least associated with cortical atrophy, significantly enriched terms included structural cellular components.

## Discussion

We mapped structural brain abnormalities in PD and contextualized this spatial pattern using connectomics, annotation enrichment and imaging transcriptomic approaches. Widespread reductions in cortical thickness, cortical surface area and subcortical volumes were found in PD relative to HCs. Regional cortical thickness and subcortical volume reductions were most sensitive to measures of advancing disease stage, longer disease duration and cognitive decline. We then showed that the pattern of PD-related cortical atrophy was constrained by network architecture. Network-based epicentres were identified in the precuneus, temporal lobes, and amygdala, which generalized across disease stages and co-localized to default mode and limbic networks in individual patients. We also found that cortical thickness and surface area deficits mapped onto specific brain systems related to distinct intrinsic networks, cytoarchitectonic tissue classes, and neurotransmitter receptor profiles. Finally, we demonstrated that the pattern of cortical atrophy correlated with gene expression profiles implicated in synaptic and neuronal processes.

Structural brain abnormalities in PD were characterized by widespread reductions in cortical thickness, cortical surface area, and subcortical volume. These findings reproduce–in an updated sample–the atrophy pattern first reported by Laansma *et al.*^3^ However, our findings of morphometric differences in PD were generally inconsistent with previous case-control studies in terms of location and size of effects.^60^ These discrepancies likely stem from limited sample sizes, clinical heterogeneity, and variability in analytical methods used in previous studies. We address these limitations by harmonizing data across multiple sites to create a large, well-powered sample and applying standardized analysis methods. The resulting atrophy pattern provides a map against which future smaller PD studies can be benchmarked. Among the structural brain metrics, cortical thickness and subcortical volume deviations were most strongly correlated with clinical features, including more advanced disease stages, longer disease durations, and poorer global cognition. This relationship between PD atrophy and measures of disease severity and cognitive impairment are well-established in the literature.^3,61,62^ Critically, our results are based on a significantly larger sample size spanning a wider disease course than most previous work.

We estimated PD-related brain changes through a *w*-scoring approach that describes the deviation of patient measures from a normative distribution derived from HCs. Although there was an imbalance in sample sizes between our PD (*n* = 3096) and HC (*n* = 1262) groups, a sufficient number of HCs was available to yield stable and representative estimates of the normative distribution. Prior work has demonstrated that such a sample size achieves reasonable and near-plateau normative modelling performance.^63^ Additionally, the standard deviation used to calculate *w*-scores in patients with PD was based on the residual error in HCs after accounting for covariates age and sex (and intracranial volume, when appropriate), which reflects the variability in the normative data. Critically, the distribution of covariates in HCs adequately covered that of patients with PD (see the ‘Results’ section).

Nodal atrophy was correlated with neighbourhood atrophy such that regions with greater atrophy were more strongly connected to neighbours with collectively greater atrophy themselves. This relationship was best captured when normative connectivity profiles were defined by structural cortico-cortical network models and was consistent across disease stages. Node-neighbourhood correlations were notably stable among single-subject atrophy maps, suggesting network spreading as a fundamental underlying mechanism in PD. Although cross-sectional by design, our approach captures the spatial embedding of pathology across the connectome, allowing for inferences about a propagating process whereby pathology accumulates in structurally and functionally connected regions. Our results extend prior findings in prodromal and *de novo* PD^1,12-17,64^ to the entire disease course and within individual patients. Similar network spreading processes have also been demonstrated in other neurodegenerative diseases and psychiatric disorders, including Alzheimer’s disease, frontotemporal dementia, amyotrophic lateral sclerosis, and schizophrenia.^6,20,46,47,65-67^ This repeated observation is consistent with the theory that neurodegeneration is in part driven by synaptic transmission of pathogenic agents, with alpha-synuclein identified as the causative protein in PD.^7,8^

In contrast to our cortical-driven results, subcortical atrophy patterns were poorly explained by subcortico-cortical network models. This lack of an association might be explained by the subcortical atlas used in this study, which features a limited number of coarsely parcellated regions, an unavoidable limitation of the ENIGMA dataset. Limited granularity in the subcortex may not adequately capture the spatial variance of PD pathology across substructures.^68,69^ Likewise, the subcortico-cortical connectivity profiles generated with this parcellation might fail to capture the spatial gradients of connectivity between the subcortex and cortex.^70-73^ Future studies should use atlases with multiple spatial resolutions to test dependency of results on spatial scale information. Alternatively, distinct connectivity profiles between cortex versus subcortex might explain their differential ability to inform patterns of disease pathology. Whereas cortical connectivity is highly distributed and hierarchical with many interconnections, subcortical connectivity is instead modular and specialized with unidirectional relay connections.^74^ Consequently, MRI-derived cortico-cortical networks may better reflect the widespread atrophy patterns in PD compared to subcortico-cortical networks.

Connectome-based atrophy modeling also allowed the identification of cortical disease epicentres, here thought to represent regions that may act as efficient propagators of pathology. At the group level, the precuneus, lateral temporal cortex, and amygdala emerged as network-based epicentres, displaying high convergence across disease stages. The precuneus and lateral temporal cortex are central hubs of the default mode network, exhibiting widespread connections with other brain areas.^75-77^ Such connectivity profiles well-position these regions as propagators of disease pathology. We also identified patient-specific epicentres: although we observed considerable regional variability in individualized epicentres, they broadly mapped to the default mode and limbic networks, and to subcortical nuclei. Importantly, the epicentres thus defined are not necessarily the originators of pathology, which are likely located outside the cerebral cortex.^1^ For example, according to the alpha-Synuclein Origin site and Connectome model,^78^ the amygdala and olfactory bulb are important origin sites of pathology in a brain-first subtype of PD whereas pathology originates from the enteric nervous system and lower brainstem in the body-first subtype. Overall, however, our findings support the robust contribution of default mode, limbic, and subcortical regions, along with their associated network architecture, in shaping the atrophy pattern in PD.

Cortical thickness abnormalities in PD mapped predominantly onto the default mode network, which aligns with our finding of individualized epicentres co-localizing to this specific network. Previous studies have also reported cortical thinning in key regions of the default mode network, such as the precuneus and posterior cingulate cortex, suggesting a consistent pattern of vulnerability in PD.^61,79,80^ These structural changes are complemented by findings from functional MRI studies in PD that have demonstrated altered connectivity within the default mode network.^81-83^ While the motor symptoms of PD are mainly caused by substantia nigra dopamine neuron loss, default mode network dysfunction likely accounts for the cognitive and mood symptoms that are a hallmark of later disease stages.^84-87^ Overall, these findings suggest that cortical PD pathology primarily affects higher-order, transmodal areas compared to unimodal cortex.

It is notable that the spatial distribution of cortical atrophy within the default mode network and temporal regions in PD appears to align with canonical tau deposition patterns observed with PET imaging in Alzheimer’s disease,^88^ as well as post-mortem studies of tau and beta-amyloid in PD and dementia with Lewy bodies.^89^ This overlap raises the possibility of Alzheimer’s disease co-pathology in PD that contributes to neurodegeneration and cognitive impairment, which is a hallmark of advanced PD.^90^ Although our modelling framework is agnostic to the precise underlying proteinopathy, we cannot rule out the potential role of tau and amyloid accumulation—alongside alpha-synuclein—to the cortical atrophy observed in PD.

We also found greater cortical abnormalities in regions with lower normative expression of specific serotonergic, histaminergic, and cholinergic neurotransmitter systems. The 5-HT1B receptor has previously been attributed a neuroprotective role, as serotonin receptor agonism reduced alpha-synuclein deposition and oxidative stress in a rodent model of PD.^91^ Similarly, the A4B2 nicotinic acetylcholine receptor mediates the neuroprotective effects of nicotine against PD pathology.^92^ Thus, regions with lower expression of these neuroreceptor systems could exhibit greater susceptibility to neurodegeneration, but further investigation is needed. In isolation, the contribution of individual receptor and transporter expression appears modest. It is plausible, however, that no single molecular variable predicts brain atrophy; rather, the collective small influence of multiple variables likely contributes to local vulnerability. Furthermore, our results suggest that other local features, such as alpha-synuclein levels^93^ or cell type composition,^19^ may also play an important role in conferring local vulnerability. Our findings, while limited to the variables available, offer an initial step towards modelling brain atrophy in PD as a product of both distributed network and local features.

Cortical atrophy in PD was associated with gene expression profiles enriched for synaptic and neuronal terms, suggesting a link between disease pathology and underlying biological mechanisms. Our finding replicates previous imaging transcriptomic studies in PD, demonstrating a link between grey matter atrophy or iron accumulation and genes related to synaptic transmission and signalling.^13,23^ Alpha-synuclein protein normally plays an important role in the regulation of synaptic function. In PD, misfolded, pathogenic alpha-synuclein has been shown to aggregate in presynaptic terminals, disrupting neurotransmission and eventually leading to neuronal death.^94-96^ This loss of neuropil;the synapses, axons, and dendrites within cortical columns;would be reflected in cortical thinning.^97^ In summary, the overexpression of genes related to synapses and neuropil components appears to identify cortical regions with heightened vulnerability to PD pathology.

We examined the contributions of network structure and local vulnerability to the pattern of atrophy in PD independently; however, it is important to consider that these biological principles are not mutually exclusive but rather interactive. In other words, the spread of pathology might be constrained to the connectivity between regions, but its impact on regional morphometry or function may be modulated by local features. This notion is demonstrated by agent-based simulations of pathology spread in PD.^22,98,99^ Zheng *et al.*^98^ used a susceptible-infected-removed model to simulate the propagation of alpha-synuclein along a structural connectome anchored by regional expression of genes that modify pathogenic protein levels. They showed that this approach could successfully recreate *in silico* the pattern of brain atrophy observed in PD *in vivo*. In short, the current study is consistent with PD pathology being the result of an interaction between connectivity and local vulnerability.

The current report has some limitations. First, the cross-sectional nature of the dataset does not consider individual variability in disease progression over time. In addition, this study relied on retrospective data collection that resulted in inconsistent availability of clinical information across the contributing sites, limiting our ability to deeply phenotype our participants. For example, the Unified Parkinson’s Disease Rating Scale is the gold-standard assessment of motor symptom severity in PD.^100^ Depending on the protocol at a given study site, this scale was administered when the PD patient was either ON or OFF their dopaminergic medications, or both ON and OFF in separate sessions, making it challenging to harmonize and interpret the resulting scores. Moreover, our analyses relied on normative models and estimates of network connectivity, gene expression, and other brain features derived from unrelated cohorts of young healthy adults to contextualize the atrophy pattern measured in PD. Relating individual patient multimodal brain data instead could perhaps better account for between-subject variability. Finally, structural brain estimates were parcellated using the Desikan-Killiany atlas^29^ in accordance with standardized ENIGMA protocols. This brain parcellation features large and heterogeneously sized regions that might bias results, although it has been previously demonstrated that different parcellations and parcellation resolutions have minimal impact on the performance of spatial null testing.^36^ Nevertheless, future work should aim to replicate the findings reported here at multiple spatial scales.

## Conclusion

In summary, we found widespread patterns of brain atrophy in PD that were shaped by network architecture, across disease stages, and in individual patients. Cortical abnormalities overlapped with maps of local brain features, including intrinsic networks, cytoarchitectonics, and neurotransmitter systems. The observed atrophy pattern also correlated with gene expression profiles related to synaptic structure and function. Our results demonstrate how we can contextualize the spatial pattern of atrophy using multimodal data to better understand the contribution of network spreading and local vulnerability in PD.

## Supplementary Material

awaf432_Supplementary_Data

## Data Availability

Publicly available datasets used in this report include the Parkinson Progression Marker Initiative (PPMI; ppmi-info.org), OpenNeuro Japan including Udall cohort (openneuro.org/datasets/ds000245), and Neurocon and Tao Wu’s datasets (fcon_1000.projects.nitrc.org/indi/retro/parkinsons.html). Individual ENIGMA-PD sites retain ownership of their MRI data and only share anonymized derived data for analysis. Data are therefore not openly available but researchers are invited to join the ENIGMA-PD working group where they can formally request derived data via secondary proposals. Data requests are then considered by the individual site’s principal investigator(s). If you are interested in joining the ENIGMA-PD, please contact enigma-pd@amsterdamumc.nl. For more information, please see the working group website: https://enigma.ini.usc.edu/ongoing/enigma-parkinsons/. Tools for mapping cortical parcellations to network and cytoarchitectonic partitions and for generating spatial null models are available as part of *netneurotools*, available at https://netneurotools.readthedocs.io/en/latest/. Structural and functional cortico-cortical and subcortico-cortical connectivity matrices are available as part of the *enigmatoolbox*, available at https://enigma-toolbox.readthedocs.io/en/latest/. Volumetric radiotracer maps of neurotransmitter receptors and transporters are available at https://github.com/netneurolab/hansen_receptors. Post-mortem gene expression data from the Allen Human Brain Atlas are available as part of the *abagen* toolbox at https://abagen.readthedocs.io/en/stable/.
