# Supplementary material for: Global network and local vulnerabilities underlie brain atrophy across Parkinson’s disease stages
Source: Brain. Author manuscript; Available in PMC 2026 Jul 30. (PMC13337226; doi:10.1093/brain/awaf432)
Supplement: Supplementary Material [file NIHMS2182552-supplement-Supplementary_Material.pdf]

Table S1: Demographic and clinical details of each ENIGMA-PD contributing site

Site	N cohorts	N participants		Age (mean ± SD)		Sex (% female)		Time since diagnosis (mean ± SD)		MoCA (mean ± SD)	
		HC	PD	HC	PD	HC	PD	HC	PD	HC	PD
Amsterdam	3	72	203	59.38 ± 9.73	63.35 ± 9.04	40.28	37.44	-	3.60 ± 3.61	28.23 ± 1.48	26.23 ± 2.17
Bern	2	49	53	62.88 ± 8.68	63.81 ± 8.76	53.06	54.72	-	12.49 ± 4.38	-	23.00 ± 5.66
Brisbane	2	-	86	-	62.55 ± 8.80	-	30.23	-	8.66 ± 4.76	-	25.91 ± 2.58
Campinas	1	134	107	59.00 ± 7.99	60.36 ± 9.74	61.94	33.64	-	7.36 ± 6.46	-	-
Chang Gung	1	223	315	60.95 ± 7.28	60.93 ± 8.44	53.81	43.49	-	8.76 ± 6.34	-	-
Charlottesville	3	-	164	-	64.30 ± 8.75	-	25	-	9.12 ± 4.70	-	24.57 ± 3.70
Christchurch	1	39	208	67.54 ± 8.53	69.38 ± 7.75	33.33	26.44	-	5.75 ± 5.57	28.05 ± 1.43	23.57 ± 4.19
Donders	2	77	516	60.79 ± 9.74	62.03 ± 8.50	46.75	42.05	-	2.87 ± 1.93	27.70 ± 1.60	26.81 ± 2.48
Graz	2	124	121	63.40 ± 10.06	63.29 ± 10.13	27.42	27.27	-	4.57 ± 4.97	-	-
Liege	2	74	72	65.36 ± 6.91	66.49 ± 7.57	47.3	41.67	-	6.47 ± 4.63	-	-
Milan	1	10	44	53.30 ± 10.53	57.77 ± 7.71	70	31.82	-	11.42 ± 3.38	-	-
Montreal	1	64	200	64.44 ± 9.99	65.52 ± 9.06	64.06	33.5	-	4.91 ± 4.53	26.16 ± 2.66	25.11 ± 3.60
NW-England	2	40	40	68.30 ± 7.25	68.75 ± 8.43	47.5	20	-	7.11 ± 4.52	27.73 ± 2.10	25.11 ± 4.01
Neurocon	1	15	27	66.73 ± 11.74	68.70 ± 10.55	80	37.04	-	-	26.14 ± 3.72	25.33 ± 2.52
ON Japan	1	15	29	63.33 ± 5.25	67.83 ± 6.77	53.33	58.62	-	-	-	-
Oxford	1	56	113	65.64 ± 8.31	64.35 ± 9.86	39.29	35.4	-	2.27 ± 1.59	27.96 ± 1.44	26.35 ± 2.80

PPMI	21	154	333	61.58 ± 10.05	62.47 ± 8.81	35.71	34.83	-	0.58 ± 0.56	28.25 ± 1.10	27.13 ± 2.27
Pennsylvania	1	12	121	70.17 ± 5.59	66.44 ± 7.79	58.33	31.4	-	7.27 ± 5.31	-	25.35 ± 3.49
Rome SLF	1	39	232	49.92 ± 6.21	63.43 ± 9.50	43.59	37.5	-	4.98 ± 4.20	-	-
Stanford	1	11	44	65.82 ± 6.46	68.61 ± 8.50	81.82	50	-	5.62 ± 3.44	27.91 ± 1.58	23.93 ± 5.21
Tao Wu	1	20	19	64.75 ± 5.58	65.00 ± 4.45	40	47.37	-	5.32 ± 4.00	-	-
Udall	1	16	24	62.56 ± 9.98	66.08 ± 10.02	61.11	28	-	8.93 ± 4.86	27.83 ± 1.47	26.40 ± 2.12
UCSF	1	18	25	66.00 ± 6.35	63.38 ± 7.51	31.25	37.5	-	-	27.67 ± 1.63	27.17 ± 1.99

HC = healthy controls; PD = Parkinson’s disease; SD = standard deviation; MoCA = Montreal Cognitive Assessment

Table S2: MRI scanning protocols and participant inclusion/exclusion criteria of each ENIGMA-PD contributing site

Site	Cohort	Diagnostic criteria	Time between MRI and clinical assessment	MRI acquisition details	PD	HC
Amsterdam	Amsterdam 1	UKBB	Same day	GE Discovery (3T); Sagittal 3-dimensional gradient-echo T1-weighted sequence (256×256 matrix; FOV = 25cm; voxel size = 1×0.98×0.98 mm; TR = 7.8 ms; TE = 3.0 ms; FA = 12°)	Inclusion: consecutive patients seen at the movement disorders outpatient clinic.<br><br>Exclusion: -	No controls
	Amsterdam 2				Inclusion: Subjective cognitive complaints (PD-CFRS > 3), HY stage < 4.<br><br>Exclusion: dementia (SAGE <14 or MoCA < 22), drugs or alcohol abuse (CAGE AID > 1), depressive symptoms (BDI > 18), impulse control disorder (ICD criteria interview), psychotic symptoms (SAPS-PD criteria), tumors and significant vascular abnormalities.	Inclusion: sex, age, and education matched.<br><br>Exclusion: neurological disease, indication of dementia (MoCA < 22), indication of psychotic (SAPS-PD) or depressive disorder (BDI > 18), drugs and/or alcohol abuse, inability to undergo neuropsychological assessment, traumatic brain injury, tumor or vascular abnormalities.
	Amsterdam 3				Inclusion: early stage, non-demented PD patients who were not using dopamine replacement therapy.<br><br>Exclusion: current psychiatric or neurological disorders other than PD, a Beck Depression Inventory (BDI) score>15 and a Mini Mental State Examination (MMSE) score <24.	Inclusion: sex, age, education, and handedness matched.<br><br>Exclusion: current psychiatric or neurological disorders, a Beck Depression Inventory (BDI) score >15 and a Mini Mental State Examination (MMSE) score<24.

Bern	BE 1	UKBB	Within 7 days	Siemens Verio (3T); MDEFT sequence (1mm^3 isotropic voxel; TR = 7.92 ms, TE = 2.48 ms, TI = 910 ms)	Inclusion: PD and familial forms of typical Parkinsonian syndromes, motor complications of dopaminergic medication that are at least moderately bothersome to the patient.<br><br>Exclusion: age > 85 years, surgical or medical contraindications for a deep brain stimulation (DBS)-implantation, severe medical illness, severe personality disorder, dementia (DSM-V criteria and MMSE < 20, current psychosis, ongoing major depression (BDI-II > 23) or depression of any severity with suicidal ideation.	Inclusion: sex and age matched.<br><br>Exclusion: -
	BE 2			Siemens Trio Tim (3T); as above		
Brisbane	Brisbane 1	UKBB	<2 days	GE (3T) Axial 3D SPGR, TE: Minimum; TI: 450 ms; Flip angle = 15; Slices 166; Voxels 256×256; slice thickness 1mm isotropic.	Inclusion: PD patients were being assessed for STN-DBS, and were at Hoehn and Yahr stage 2 or greater with motor fluctuations or other motor complications related to dopaminergic therapy.<br><br>Exclusion: Mini-Mental State Examination Score (MMSE) of <25 or a clinical diagnosis of PD dementia according to published Movement Disorder Society criteria.	No controls.
	Brisbane 2			Siemens Prisma (3T) 3D T1-weighted MPRAGE; TR = 2000 ms; TE = 2.8 ms; TI = 929 ms; Axial acquisition; 196 slices; Vo×els 256x256; Voxel size 1mm isotropic.	As above	As above

Campinas	UNICAMP	UKBB	15.3 days on average (SD=11.1)	Philips Achieva (3T); 3D T1-weighted image acquired on the sagittal plane (FOV of 240×240mm; 1mm^{3}isotropic voxel, TR = 7ms, TE = 3.2ms; FA = 8°)	Inclusion: idiopathic PD, taking antiparkinsonian medications, age > 30 years.<br><br>Exclusion: clinically significant musculoskeletal, cardiovascular, respiratory or other neurological disease.	Inclusion: age > 30 years old.<br><br>Exclusion: clinically significant musculoskeletal, cardiovascular, respiratory or neurological disease.
Chang Gung	CGU	NINDS	Within 30 days, except for one participant (45 days)	Siemens Magnetom TrioTim (3T); T1-weighted images were acquired using an MPRAGE (224×256 matrix; FOV = 224 mm×256 mm; 1mm^3 isotropic voxel; TE = 2.63ms; TR = 2000 ms, FA = 9°)	Inclusion: diagnosis of probable PD, ability to tolerate treatment discontinuation for 12 hours.<br><br>Exclusion: major physical illnesses, psychiatric disorders, known brain abnormalities, history of intracranial surgery, pharmacotherapy for more than ten years or treatment with drugs able to cross the blood-brain-barrier (other than those used to treat PD).	Inclusion: aged between 50- 90.<br><br>Exclusion: same as in PD.
Charlottesville	Charlottesville 1<br>Charlottesville 2<br>Charlottesville 3	PD diagnosis confirmed by neurologist	73.9 days on average	Siemens (3T); Stock MPRAGE. Acquisition parameters vary by scanner protocol. Voxel size varied but did not exceed 1×1×1.2 mm.	Inclusion: PD diagnosis with a motor symptom that is not (or inconsistently) responsive to oral medication. Exclusion: -	No controls

Christchurch	Christchurch	UKBB	28 days on average (SD=48)	General Electric HDx (3T); SPGR sequence	Inclusion: met the UK Parkinson's Society criteria for PD, motor symptoms present for at least 1 year at study entry.<br><br>Exclusion: atypical parkinsonian disorder, history of moderate/severe head injury, stroke, early-life learning disability, major psychiatric or medical illness in the previous 6 months, poor English (precluding testing).	Inclusion: -<br><br>Exclusion: neurological disease/disorder; history of moderate/severe head injury, stroke, early-life learning disability, major psychiatric or medical illness in the previous 6 months, poor English (precluding testing).
Donders	Donders 1	UKBB	Same day	Siemens Magnetom Trio (3T); 3D T1 weighted image acquired on the sagittal plane (FOV of 256×256mm; 1mm^3 isotropic voxel, TR = 2300, TE = 3.03, TI = 1100, FA = 8°)	Inclusion: Idiopathic PD, UPDRS tremor-score > 2, dopaminergic therapy with a clear clinical response of non-tremor symptoms (bradykinesia, rigidity), HY stage 1-3. Exclusion: Neurological or psychiatric comorbidity, severe head tremor or dyskinesias, cognitive impairment (MMSE < 26), co-medication associated with elongated QT-time, pregnancy, age < 25 years.	Inclusion: same age/gender balance as PD patients. Exclusion: Neurological or psychiatric disease, cognitive impairment (MMSE < 26), medication associated with elongated QT-time, pregnancy, age < 25 years.
	Donders 2			Siemens Magnetom Prisma (3T); T1-weighted anatomical acquired using an MPRAGE sequence. 1mm^3 isotropic; FOV = 256 mm; 192 slices; TR/TE/TI = 2000/2/880 ms; FA = 8°.	Inclusion: Idiopathic PD diagnosed by a certified neurologist. 0-5 years disease duration. >18 years of age. Able to read and understand Dutch. Able to provide informed consent and comply with all aspects of the study protocol.<br>Exclusion: Co-morbidities severe enough to impair interpretation of parkinsonian disability. Contraindications to magnetic resonance imaging. Pregnancy or breastfeeding. Nickel allergy.	Same as for PD patients, with the additional exclusion criteria of having co-morbidities that would influence the interpretability of results from group comparisons with PD patients.
Graz	PROMOVE ASPS 1	QSBB	90% same day, max of 4 weeks	Siemens Magnetom Trio/Prisma (3T); PD: structural T1-weighted MPRAGE sequence (1mm^3 isotropic voxel; TR = 1900ms; TI =900ms; FA = 9°; TE: 2.19ms (101 patients) + TE: 2.7ms (23 patients) HC: structural T1-weighted	Inclusion: Clinical diagnosis of PD.<br><br>Exclusion: MMSE <24, secondary parkinsonism, atypical parkinsonian diseases, a history of neuroleptic drugs, structural abnormalities on	Inclusion: No history of previous stroke or dementia and a normal neurologic examination.<br><br>Exclusion: -
	PROMOVE ASPS 2					

				MPRAGE sequence (1mm^3 isotropic voxel; TR = 1900ms; TE = 2.19ms; TI = 900ms; FA = 9°)	routine MRI scans or a history of previous stroke.	
Liege	Liege 1<br>Liege 2	UKBB	Same day	Siemens Magnetom Allegra (3T); 3D multi-echo fast low angle shot (FLASH) sequence (256×224 matrix; 1mm^3 isotropic voxel; TR = 18.7 ms; TE = 2.2-14.7 ms; FA = 20°)	Inclusion: Non-demented PD patients.<br><br>Exclusion: -	Inclusion: age, sex, and highest achieved education level matched.<br><br>Exclusion: -
Milan	Milan	UKBB	Within 1 month	Philips Achieva (3T); 240×240 mm matrix; 1mm^3 isotropic voxel; FOV = 33.7×24 cm; TR = 9.81 ms; TE = 4.6 ms; FA =8°	Inclusion: PD diagnosis.<br><br>Exclusion: -	Inclusion: -<br><br>Exclusion: -
Montreal	Montreal	PD diagnosis confirmed by neurologist	Within 6 months	Siemens Prisma (3T); 3DT1 (sagittal MPRAGE); slice thickness = 1mm^3, TR = 2.3 s, TE = 2.98 ms, TI = 900 ms, FA = 9°	Inclusion: PD diagnosis, 18 years of age or older, and able to read and understand French or English.<br><br>Exclusion: clinical diagnosis of dementia, other movement disorder, or other serious neurological illnesses; taking neuroleptics; claustrophobia, heart implants, or other contraindications to MRI.	Inclusion: 18 years of age or older and able to read and understand French or English.<br><br>Exclusion: clinical diagnosis of dementia or a serious neurological illness; claustrophobia, heart implants, or other contraindications to MRI.
NW-England	NW-England 1<br>NW-England 2	UKBB	Same day	Philips Achieva (3T); MPRAGE IR Method (voxel size 0.94×0.94×1 mm; FOV = 240 (AP)×192 (RL) mm, TR = 8.4 ms, TE = 3.9 ms, TI = 1150 ms, FA = 8°)	Inclusion: PD diagnosis without known clinical cardiovascular disease or dementia. No other significant neurological conditions.	Inclusion: age-matched to PD group and without a history of idiopathic PD or clinical CVD, or any other significant neurological condition.

Neurocon	Neurocon	MDS	Not available	Siemens Avanto (1.5T); MPRAGE IR Method (voxel size 0.97×0.97×1 mm; TR = 1940 ms; TE = 3.08 ms)	Inclusion: Early- or moderate stage of PD.<br><br>Exclusion: -	Inclusion: no history of neurological or psychiatric disease.
ON Japan	ON Japan	UKBB	Not available	Siemens Magnetom Verio (3T); High resolution T1-weighted images (256×256 matrix size; FOV = 256 mm; TR = 2.5 s, TE = 2.48 ms)	Inclusion: -<br><br>Exclusion: history of other neurological or psychiatric disease, focal white matter abnormalities. ACE-R score ≤ 88, psychiatric symptoms (hallucinations, depression, etc.)	Inclusion: -<br><br>Exclusion: neurological disease, family history of PD, or hyposmia, and with an ACE-R score > 88 in the study.
Oxford	Oxford DISCOVERY	UKBB	108 days on average (SD=104)	Siemens Trio (3T); MPRAGE ($1mm^3$ isotropic voxel, TE = 4.7 ms; TR = 2040 ms; TI = 900 ms; FA = 8°)	Inclusion: PD diagnosis within the past 3.5 years. Full details of criteria are available at: Szewczyk-Krolikowski K et al. (2013). No atypical features to suggest an alternative diagnosis.<br><br>Exclusion: secondary parkinsonism due to head trauma or medication use, atypical parkinsonism syndromes (multiple system atrophy, progressive supra nuclear palsy, corticobasal degeneration, dementia with Lewy bodies), documented postural BP drop on standardized measurement or significant urinary symptoms.	Inclusion: controls without blood relatives with PD.

PPMI	PPMI 1-21	MDS	Same day	Siemens Trio Tim (3T); T1-3D e.g. MPRAGE, SPGR, Sagittal (56×256×170-200 matrix; slice thickness = 1.2 mm; voxel size = 1×1×1.2 mm)	Inclusion & exclusion criteria detailed here: www.ppmi-info.org/wp-content/uploads/2013/02/PPMI-Protocol-AM5-Final-27Nov2012v6-2.pdf	Inclusion & exclusion criteria detailed here: www.ppmi-info.org/wp-content/uploads/2013/02/PPMI-Protocol-AM5-Final-27Nov2012v6-2.pdf.
Pennsylvania	Pennsylvania	UKBB	MoCA: 53.7 days on average (SD=67.1) HY: 51.4 days on average (SD=65.0)	Siemens Trio/Prisma (3T); 3D MPRAGE Sagittal & Axial (slice thickness = 1 mm; TR = 1620/1800/2300 ms; TE = 2.95/3.8/3.09 ms; TI = 900/950 ms)	Inclusion: clinical diagnosis of PD.<br>Exclusion: -	Inclusion: > 40 years of age, MMSE > 27, a negative self-reported history of neurological or psychiatric condition, and MRI safe (e.g., no metal, claustrophobia).<br>Exclusion: -
Rome SLF	Rome SLF	MDS	1 day	Siemens Allegra (3T); T1MDEFT (256×224 matrix; 1mm^3 isotropic voxel; TR = 7.92 ms; TE = 2.4 ms; FA = 15°)	Inclusion: diagnosis of idiopathic, MMSE score > 26, no dementia.<br>Exclusion: presence of major non-stabilized medical, known or suspected history of alcoholism, drug dependence and abuse, head trauma, and mental disorders (apart from mood or anxiety disorders, history of neurological diseases other than idiopathic PD, unclear history of chronic dopaminergic treatment responsiveness.	Inclusion: vision and hearing sufficient for compliance with testing procedures, laboratory values within normal reference intervals, neuropsychological domain scores above normal cognitive level cutoff scores, corrected for age and educational level.<br>Exclusion: dementia or MCI diagnosis, confirmed by a comprehensive neuropsychological battery, MMSE score < 26, presence of major non stabilized medical illnesses, known or suspected history of alcoholism, drug dependence and abuse, head trauma, and mental disorders (apart from mood or anxiety disorders).

Stanford	Stanford	UKBB	Within 3 months	General Electric SIGNA (3T); FSPGR 3D T1 scan	Inclusion: > 20% improvement on MDS-UPDRS part III ON meds compared to OFF meds.<br><br>Exclusion: -	Inclusion: normal neurological exam and normal neuropsychiatric battery (within 1.5 SD of age- and education-adjusted norms).<br><br>Exclusion: -
Tao Wu	Tao Wu	MDS	1-2 days	Siemens Magnetom Trio (3T); MPRAGE IR method (1mm^3 isotropic voxel; TR = 1100ms; TE =3.39ms)	Inclusion: diagnosis of PD based on medical history, physical and neurological examinations, response to levodopa or dopaminergic drugs, and laboratory tests and MRI scans to exclude other diseases.<br><br>Exclusion: -	Inclusion: -<br><br>Exclusion: -
UCSF	UCSF	MDS	1-2 days	Siemens Skyra (3T) 3D T1-weighted MPRAGE; TR (ms) = 2300; TE (ms) = 3; TI (ms) = 1000; Flip Angle 9°; Acquisition plane: Sagittal; Number of Slices: 256; FOV = 256×256; Voxel size (mm^3) = 1×1×1	Inclusion: Ages 40 to 85; Healthy or Diagnosis of Parkinson's disease and mild to moderate symptoms defined by a Hoehn and Yahr score of 1.0 to 3.0; Diagnosis of a movement disorder with Parkinsonian symptoms, including but not limited to multiple system atrophy.<br><br>Exclusion: Any contra-indication for undergoing MRI, including pregnancy, pacemakers, or other implanted electronic electric devices. Subjects may also be excluded for having various types of metal in their bodies. This will be evaluated by trained staff on a case-by-case basis. claustrophobia that would cause the subject difficulty abnormal MRI findings, such as severe cortical or subcortical atrophy, brain tumors, major vascular disease; prior brain surgery; alcohol or other substance abuse; history of encephalitis, multiple sclerosis, other CNS infection, epilepsy, or primary CNS disease besides PD, loss of consciousness for more than 2	Inclusion:<br><br>Exclusion: Any contra-indication for undergoing MRI, including pregnancy, pacemakers, or other implanted electronic electric devices. Subjects may also be excluded for having various types of metal in their bodies. This will be evaluated by trained staff on a case-by-case basis. claustrophobia that would cause the subject difficulty abnormal MRI findings, such as severe cortical or subcortical atrophy, brain tumors, major vascular disease; prior brain surgery; alcohol or other substance abuse; history of encephalitis, multiple sclerosis, other CNS infection, epilepsy, or primary CNS disease besides PD, loss of consciousness for more than 2 minutes; clinical diagnosis of dementia, as indicated by clinical interview (mild cognitive impairment will be accepted); ongoing severe hallucinations; prior exposure to neuroleptic agents; Inability to give informed consent.

					minutes; clinical diagnosis of dementia, as indicated by clinical interview (mild cognitive impairment will be accepted); ongoing severe hallucinations; prior exposure to neuroleptic agents; Inability to give informed consent.	
Udall	Udall	Not available	Not available	Philips Achieva (3T); sagittal T1-weighted 3D MPRAGE (176 slices, matrix size = 256×256, inversion time = 1100 ms, turbo-field echo factor = 225, TR = 7.46 ms, TE = 3.49 ms, flip angle = 7°, shot interval = 2530 ms) with 1 mm isotropic voxels	Inclusion: -<br><br>Exclusion: potential participants were excluded if they had a history of any primary neurodegenerative disease other than idiopathic PD, brain surgery (including placement of a deep brain stimulator), moderate to severe dyskinesia, significant head trauma, stroke history, severe or unstable cardiovascular disease, contraindications to MRI, or a Montreal Cognitive Assessment score (MoCA) (Nasreddine et al., 2005) < 23.	Not available

PD = Parkinson's disease; HC = healthy controls; TR = repetition time; TE = echo time; TI = inversion time; FOV = field of view; FA = flip angle; UKBB = UK Biobank; MDS = Movement Disorders Society; QSBB = Queen Square Brain Bank; NINDS = National Institute of Neurological Disorders and Stroke

Table S3: Partial R^2 of cohort in unadjusted versus ComBat-adjusted cortical thickness

Region	Unadjusted data		ComBat-adjusted data	
	LH	RH	LH	RH
bankssts	0.28	0.28	0.00	0.00
caudalanteriorcingulate	0.18	0.18	0.00	0.00
caudalmiddlefrontal	0.23	0.17	0.00	0.00
cuneus	0.29	0.51	0.00	0.00
entorhinal	0.23	0.58	0.00	0.00
fusiform	0.35	0.62	0.00	0.00
inferiorparietal	0.27	0.36	0.00	0.00
inferiortemporal	0.35	0.48	0.00	0.00
isthmuscingulate	0.19	0.30	0.00	0.00
lateraloccipital	0.25	0.25	0.00	0.00
lateralorbitofrontal	0.28	0.48	0.00	0.00
lingual	0.28	0.52	0.00	0.00
medialorbitofrontal	0.20	0.42	0.00	0.00
middletemporal	0.27	0.40	0.00	0.00
parahippocampal	0.19	0.21	0.00	0.00
paracentral	0.34	0.37	0.00	0.00
parsopercularis	0.25	0.25	0.00	0.00
parsorbitalis	0.15	0.22	0.00	0.00
parstriangularis	0.25	0.28	0.00	0.00
pericalcarine	0.36	0.63	0.00	0.00
postcentral	0.33	0.50	0.00	0.00
posteriorcingulate	0.20	0.44	0.00	0.00
precentral	0.30	0.27	0.00	0.00
precuneus	0.31	0.31	0.00	0.00
rostralanteriorcingulate	0.17	0.38	0.00	0.00

rostralmiddlefrontal	0.28	0.53	0.00	0.00
superiorfrontal	0.21	0.40	0.00	0.00
superiorparietal	0.32	0.46	0.00	0.00
superiortemporal	0.25	0.57	0.00	0.00
supramarginal	0.25	0.31	0.00	0.00
frontalpole	0.12	0.22	0.01	0.00
temporalpole	0.15	0.44	0.00	0.00
transversetemporal	0.24	0.68	0.00	0.00
insula	0.18	0.45	0.00	0.00

LH = left hemisphere; RH = right hemisphere

Table S4: Partial R^2 of cohort in unadjusted versus ComBat-adjusted cortical surface area

Region	Unadjusted data		ComBat-adjusted data	
	LH	RH	LH	RH
bankssts	0.11	0.16	0.00	0.00
caudalanteriorcingulate	0.11	0.15	0.00	0.00
caudalmiddlefrontal	0.12	0.07	0.00	0.00
cuneus	0.09	0.08	0.00	0.00
entorhinal	0.07	0.06	0.00	0.00
fusiform	0.15	0.18	0.00	0.00
inferiorparietal	0.13	0.13	0.00	0.00
inferiortemporal	0.13	0.11	0.00	0.00
isthmuscingulate	0.10	0.14	0.00	0.00
lateraloccipital	0.13	0.12	0.00	0.00
lateralorbitofrontal	0.11	0.13	0.00	0.00
lingual	0.13	0.14	0.00	0.00
medialorbitofrontal	0.15	0.18	0.00	0.00
middletemporal	0.12	0.14	0.00	0.00
parahippocampal	0.11	0.11	0.00	0.00
paracentral	0.08	0.11	0.00	0.00
parsopercularis	0.10	0.07	0.00	0.00
parsorbitalis	0.12	0.10	0.00	0.00
parstriangularis	0.06	0.06	0.00	0.00
pericalcarine	0.09	0.10	0.00	0.00
postcentral	0.10	0.08	0.00	0.00
posteriorcingulate	0.08	0.13	0.00	0.00
precentral	0.10	0.12	0.00	0.00
precuneus	0.11	0.12	0.00	0.00
rostralanteriorcingulate	0.10	0.21	0.00	0.00

rostralmiddlefrontal	0.16	0.15	0.00	0.00
superiorfrontal	0.18	0.18	0.00	0.00
superiorparietal	0.10	0.13	0.00	0.00
superiortemporal	0.11	0.11	0.00	0.00
supramarginal	0.11	0.10	0.00	0.00
frontalpole	0.33	0.17	0.01	0.00
temporalpole	0.09	0.14	0.00	0.00
transversetemporal	0.08	0.07	0.00	0.00
insula	0.28	0.19	0.00	0.00

LH = left hemisphere; RH = right hemisphere

Table S5: Partial R^2 of cohort in unadjusted versus ComBat-adjusted subcortical volume

Region	Unadjusted data		ComBat-adjusted data	
	LH	RH	LH	RH
lateral ventricle	0.05	0.05	0.00	0.00
thalamus	0.28	0.24	0.00	0.00
caudate	0.15	0.22	0.00	0.00
putamen	0.21	0.17	0.00	0.00
pallidum	0.43	0.44	0.00	0.00
hippocampus	0.18	0.16	0.00	0.00
amygdala	0.15	0.23	0.00	0.00
accumbens	0.30	0.21	0.00	0.00

LH = left hemisphere; RH = right hemisphere

Table S6: Sample characteristics of each cross-validation fold

Fold	N of Training Set	Age	Sex (% female)	HY disease stage
1	2476	63.71	36.23	2.05
2	2477	63.81	36.21	2.05
3	2477	63.60	36.58	2.05
4	2477	63.70	35.61	2.05
5	2477	63.62	36.90	2.05

Table S7: Demographics of the age- and sex-matched subsample

	PD	HC	SMD
**N**	2555	867	-
**Age (mean ± SD)**	63.69 (9.09)	62.94 (9.33)	0.08
**Sex (% female)**	0.37	0.43	0.12

PD = Parkinson's disease; HC = healthy controls; SD = standard deviation; SMD = standard mean difference

Table S8: One-sample *t*-test of cortical thickness *w*-score estimates

Region	Mean	SD	T-stat	P-val	P_{FDR}
left bankssts	-0.259	1.042	-13.858	<0.001	<0.001
left caudalanteriorcingulate	0.071	1.030	3.827	<0.001	<0.001
left caudalmiddlefrontal	-0.179	1.074	-9.291	<0.001	<0.001
left cuneus	-0.113	1.012	-6.203	<0.001	<0.001
left entorhinal	-0.170	1.052	-9.006	<0.001	<0.001
left fusiform	-0.314	1.089	-16.039	<0.001	<0.001
left inferiorparietal	-0.282	1.090	-14.410	<0.001	<0.001
left inferiortemporal	-0.336	1.086	-17.227	<0.001	<0.001
left isthmuscingulate	-0.217	1.010	-11.978	<0.001	<0.001
left lateraloccipital	-0.214	1.033	-11.514	<0.001	<0.001
left lateralorbitofrontal	-0.142	1.088	-7.248	<0.001	<0.001
left lingual	-0.156	1.018	-8.550	<0.001	<0.001
left medialorbitofrontal	-0.155	1.042	-8.275	<0.001	<0.001
left middletemporal	-0.327	1.034	-17.605	<0.001	<0.001
left parahippocampal	-0.183	1.020	-9.999	<0.001	<0.001
left paracentral	-0.143	1.149	-6.941	<0.001	<0.001
left parsopercularis	-0.073	1.077	-3.756	<0.001	<0.001
left parsorbitalis	-0.133	1.022	-7.231	<0.001	<0.001
left parstriangularis	-0.105	1.039	-5.623	<0.001	<0.001
left pericalcarine	-0.087	1.082	-4.498	<0.001	<0.001
left postcentral	-0.125	1.055	-6.594	<0.001	<0.001
left posteriorcingulate	-0.099	1.046	-5.247	<0.001	<0.001
left precentral	-0.158	1.168	-7.521	<0.001	<0.001
left precuneus	-0.197	1.098	-9.961	<0.001	<0.001
left rostralanteriorcingulate	-0.007	1.014	-0.378	0.706	0.716
left rostralmiddlefrontal	-0.092	1.070	-4.801	<0.001	<0.001

left superiorfrontal	-0.138	1.081	-7.083	<0.001	<0.001
left superiorparietal	-0.154	1.093	-7.851	<0.001	<0.001
left superiortemporal	-0.252	1.058	-13.228	<0.001	<0.001
left supramarginal	-0.226	1.108	-11.321	<0.001	<0.001
left frontalpole	-0.027	1.009	-1.511	0.131	0.137
left temporalpole	-0.133	1.076	-6.873	<0.001	<0.001
left transversetemporal	-0.132	1.032	-7.121	<0.001	<0.001
left insula	-0.051	1.036	-2.759	0.006	0.006
right bankssts	-0.254	1.094	-12.938	<0.001	<0.001
right caudalanteriorcingulate	-0.006	1.067	-0.332	0.740	0.740
right caudalmiddlefrontal	-0.069	1.109	-3.464	0.001	0.001
right cuneus	-0.102	1.010	-5.635	<0.001	<0.001
right entorhinal	-0.204	1.047	-10.833	<0.001	<0.001
right fusiform	-0.277	1.057	-14.569	<0.001	<0.001
right inferiorparietal	-0.305	1.080	-15.707	<0.001	<0.001
right inferiortemporal	-0.299	1.056	-15.752	<0.001	<0.001
right isthmuscingulate	-0.244	1.062	-12.798	<0.001	<0.001
right lateraloccipital	-0.225	1.004	-12.496	<0.001	<0.001
right lateralorbitofrontal	-0.171	1.037	-9.193	<0.001	<0.001
right lingual	-0.148	1.006	-8.172	<0.001	<0.001
right medialorbitofrontal	-0.134	1.039	-7.174	<0.001	<0.001
right middletemporal	-0.319	1.067	-16.652	<0.001	<0.001
right parahippocampal	-0.194	1.066	-10.142	<0.001	<0.001
right paracentral	-0.063	1.115	-3.121	0.002	0.002
right parsopercularis	-0.072	1.042	-3.871	<0.001	<0.001
right parsorbitalis	-0.095	0.984	-5.399	<0.001	<0.001
right parstriangularis	-0.033	1.048	-1.764	0.078	0.084
right pericalcarine	-0.057	1.065	-2.998	0.003	0.003

right postcentral	-0.133	1.075	-6.863	<0.001	<0.001
right posteriorcingulate	-0.181	1.055	-9.562	<0.001	<0.001
right precentral	-0.096	1.138	-4.708	<0.001	<0.001
right precuneus	-0.249	1.098	-12.634	<0.001	<0.001
right rostralanteriorcingulate	-0.010	1.088	-0.536	0.592	0.610
right rostralmiddlefrontal	-0.029	1.056	-1.514	0.130	0.137
right superiorfrontal	-0.145	1.098	-7.362	<0.001	<0.001
right superiorparietal	-0.212	1.073	-10.996	<0.001	<0.001
right superiortemporal	-0.218	1.044	-11.643	<0.001	<0.001
right supramarginal	-0.255	1.102	-12.861	<0.001	<0.001
right frontalpole	-0.130	1.031	-7.026	<0.001	<0.001
right temporalpole	-0.148	1.089	-7.539	<0.001	<0.001
right transversetemporal	-0.142	1.062	-7.418	<0.001	<0.001
right insula	-0.097	1.046	-5.137	<0.001	<0.001

Table S9: One-sample *t*-test of cortical surface area *w*-score estimates

Region	Mean	SD	T-stat	P-val	P_{FDR}
left bankssts	-0.044	1.048	-2.356	0.019	0.025
left caudalanteriorcingulate	-0.005	1.008	-0.263	0.793	0.793
left caudalmiddlefrontal	-0.017	1.065	-0.873	0.383	0.407
left cuneus	-0.144	0.989	-8.087	<0.001	<0.001
left entorhinal	-0.051	1.034	-2.738	0.006	0.009
left fusiform	-0.113	1.085	-5.808	<0.001	<0.001
left inferiorparietal	-0.122	1.066	-6.387	<0.001	<0.001
left inferiortemporal	-0.104	1.071	-5.396	<0.001	<0.001
left isthmuscingulate	0.061	1.087	3.108	0.002	0.003
left lateraloccipital	-0.190	1.028	-10.289	<0.001	<0.001
left lateralorbitofrontal	-0.125	1.041	-6.698	<0.001	<0.001
left lingual	-0.164	1.033	-8.850	<0.001	<0.001
left medialorbitofrontal	-0.083	1.060	-4.337	<0.001	<0.001
left middletemporal	-0.156	1.017	-8.550	<0.001	<0.001
left parahippocampal	-0.033	1.067	-1.745	0.081	0.098
left paracentral	0.030	1.009	1.641	0.101	0.118
left parsopercularis	-0.026	1.023	-1.413	0.158	0.179
left parsorbitalis	-0.115	1.059	-6.032	<0.001	<0.001
left parstriangularis	-0.052	1.018	-2.866	0.004	0.006
left pericalcarine	-0.134	1.017	-7.350	<0.001	<0.001
left postcentral	-0.017	1.040	-0.921	0.357	0.391
left posteriorcingulate	-0.008	1.080	-0.388	0.698	0.708
left precentral	-0.037	1.037	-1.988	0.047	0.060
left precuneus	-0.138	1.021	-7.546	<0.001	<0.001
left rostralanteriorcingulate	-0.032	1.065	-1.667	0.096	0.114
left rostralmiddlefrontal	-0.151	1.067	-7.861	<0.001	<0.001

left superiorfrontal	-0.107	1.042	-5.708	<0.001	<0.001
left superiorparietal	-0.154	0.984	-8.737	<0.001	<0.001
left superiortemporal	-0.082	1.038	-4.372	<0.001	<0.001
left supramarginal	-0.089	1.051	-4.689	<0.001	<0.001
left frontalpole	-0.195	1.007	-10.761	<0.001	<0.001
left temporalpole	-0.066	1.017	-3.589	<0.001	0.001
left transversetemporal	0.073	1.053	3.851	<0.001	<0.001
left insula	-0.033	1.045	-1.768	0.077	0.095
right bankssts	-0.011	1.057	-0.598	0.550	0.576
right caudalanteriorcingulate	-0.028	1.020	-1.507	0.132	0.152
right caudalmiddlefrontal	-0.044	1.006	-2.420	0.016	0.021
right cuneus	-0.190	1.022	-10.348	<0.001	<0.001
right entorhinal	-0.086	1.005	-4.743	<0.001	<0.001
right fusiform	-0.116	1.106	-5.858	<0.001	<0.001
right inferiorparietal	-0.085	1.049	-4.497	<0.001	<0.001
right inferiortemporal	-0.100	1.037	-5.387	<0.001	<0.001
right isthmuscingulate	0.104	1.070	5.399	<0.001	<0.001
right lateraloccipital	-0.181	1.030	-9.797	<0.001	<0.001
right lateralorbitofrontal	-0.036	1.017	-1.961	0.050	0.063
right lingual	-0.186	1.001	-10.313	<0.001	<0.001
right medialorbitofrontal	-0.096	1.052	-5.098	<0.001	<0.001
right middletemporal	-0.154	1.055	-8.142	<0.001	<0.001
right parahippocampal	0.019	1.150	0.911	0.363	0.391
right paracentral	0.051	1.069	2.637	0.008	0.012
right parsopercularis	-0.047	1.056	-2.476	0.013	0.019
right parsorbitalis	-0.058	1.038	-3.087	0.002	0.003
right parstriangularis	-0.075	1.081	-3.848	<0.001	<0.001
right pericalcarine	-0.124	1.014	-6.794	<0.001	<0.001

right postcentral	-0.067	1.050	-3.531	<0.001	0.001
right posteriorcingulate	-0.046	1.028	-2.507	0.012	0.017
right precentral	0.019	1.045	1.035	0.301	0.335
right precuneus	-0.063	1.082	-3.215	0.001	0.002
right rostralanteriorcingulate	-0.100	1.047	-5.315	<0.001	<0.001
right rostralmiddlefrontal	-0.152	1.048	-8.074	<0.001	<0.001
right superiorfrontal	-0.080	1.062	-4.202	<0.001	<0.001
right superiorparietal	-0.098	1.016	-5.345	<0.001	<0.001
right superiortemporal	-0.048	1.054	-2.523	0.012	0.017
right supramarginal	-0.075	1.036	-4.026	<0.001	<0.001
right frontalpole	-0.080	1.013	-4.375	<0.001	<0.001
right temporalpole	-0.042	1.034	-2.277	0.023	0.030
right transversetemporal	0.007	1.028	0.391	0.696	0.708
right insula	-0.078	1.040	-4.146	<0.001	<0.001

Table S10: One-sample *t*-test of subcortical volume *w*-score estimates

Region	Mean	SD	T-stat	P-val	P_{FDR}
left lateral ventricle	0.132	1.167	6.292	<0.001	<0.001
left thalamus	0.087	1.085	4.438	<0.001	<0.001
left caudate	-0.120	1.113	-5.983	<0.001	<0.001
left putamen	-0.204	1.100	-10.298	<0.001	<0.001
left pallidum	-0.068	1.122	-3.353	0.001	0.001
left hippocampus	-0.143	1.078	-7.379	<0.001	<0.001
left amygdala	-0.252	1.057	-13.263	<0.001	<0.001
left accumbens	-0.133	1.041	-7.131	<0.001	<0.001
right lateral ventricle	0.173	1.201	8.033	<0.001	<0.001
right thalamus	0.038	1.092	1.957	0.050	0.054
right caudate	-0.051	1.090	-2.599	0.009	0.011
right putamen	-0.197	1.072	-10.229	<0.001	<0.001
right pallidum	-0.030	1.097	-1.542	0.123	0.123
right hippocampus	-0.153	1.086	-7.826	<0.001	<0.001
right amygdala	-0.176	1.073	-9.131	<0.001	<0.001
right accumbens	-0.133	1.078	-6.844	<0.001	<0.001

Table S11: Relationships between cortical thickness with clinical variables in PD

Region	HY disease stage			Time since diagnosis			Global cognition		
	ρ	P-val	P_{FDR}	β	P-val	P_{FDR}	β	P-val	P_{FDR}
left bankssts	-0.059	0.003	0.008	-0.007	0.060	0.091	-0.027	0.001	0.003
left caudalanteriorcingulate	0.029	0.146	0.168	0.002	0.596	0.623	0.005	0.532	0.557
left caudalmiddlefrontal	-0.040	0.045	0.063	-0.013	0.001	0.006	-0.027	0.001	0.003
left cuneus	-0.031	0.118	0.144	-0.009	0.012	0.026	-0.004	0.608	0.626
left entorhinal	-0.075	<0.001	0.001	-0.009	0.019	0.037	-0.013	0.101	0.120
left fusiform	-0.085	<0.001	<0.001	-0.016	<0.001	<0.001	-0.042	<0.001	<0.001
left inferiorparietal	-0.079	<0.001	<0.001	-0.019	<0.001	<0.001	-0.023	0.005	0.010
left inferiortemporal	-0.080	<0.001	<0.001	-0.012	0.002	0.006	-0.032	<0.001	<0.001
left isthmuscingulate	-0.066	0.001	0.003	-0.008	0.032	0.055	-0.018	0.018	0.028
left lateraloccipital	-0.049	0.014	0.025	-0.008	0.029	0.050	-0.006	0.425	0.452
left lateralorbitofrontal	-0.052	0.009	0.019	-0.007	0.099	0.137	-0.015	0.077	0.099
left lingual	-0.082	<0.001	<0.001	-0.006	0.126	0.168	-0.021	0.006	0.011
left medialorbitofrontal	-0.021	0.290	0.299	-0.004	0.275	0.323	-0.008	0.338	0.365
left middletemporal	-0.113	<0.001	<0.001	-0.012	0.001	0.006	-0.037	<0.001	<0.001
left parahippocampal	-0.056	0.004	0.011	-0.007	0.062	0.091	-0.013	0.085	0.105
left paracentral	-0.024	0.231	0.254	-0.004	0.318	0.355	-0.028	0.001	0.004
left parsopercularis	-0.071	<0.001	0.002	-0.013	0.001	0.004	-0.025	0.002	0.005
left parsorbitalis	-0.034	0.088	0.109	-0.005	0.195	0.239	-0.012	0.112	0.132
left parstriangularis	-0.046	0.020	0.031	-0.014	<0.001	0.002	-0.033	<0.001	<0.001
left pericalcarine	-0.035	0.080	0.100	-0.008	0.043	0.071	-0.017	0.043	0.058
left postcentral	-0.038	0.053	0.069	-0.010	0.009	0.020	-0.039	<0.001	<0.001
left posteriorcingulate	-0.031	0.120	0.144	-0.003	0.370	0.405	-0.011	0.165	0.184
left precentral	-0.051	0.011	0.022	-0.012	0.005	0.013	-0.042	<0.001	<0.001
left precuneus	-0.066	0.001	0.003	-0.017	<0.001	<0.001	-0.022	0.009	0.015
left rostralanteriorcingulate	-0.021	0.297	0.301	-0.004	0.314	0.355	-0.011	0.149	0.169
left rostralmiddlefrontal	-0.047	0.018	0.029	-0.011	0.007	0.016	-0.036	<0.001	<0.001

left superiorfrontal	-0.039	0.049	0.067	-0.011	0.006	0.015	-0.026	0.002	0.004
left superiorparietal	-0.053	0.007	0.018	-0.015	<0.001	0.001	-0.027	0.001	0.004
left superiortemporal	-0.097	<0.001	<0.001	-0.012	0.002	0.006	-0.037	<0.001	<0.001
left supramarginal	-0.089	<0.001	<0.001	-0.010	0.014	0.029	-0.030	<0.001	0.002
left frontalpole	-0.039	0.050	0.067	-0.004	0.275	0.323	-0.023	0.003	0.007
left temporalpole	-0.050	0.012	0.023	-0.013	0.001	0.004	-0.018	0.029	0.041
left transversetemporal	-0.066	0.001	0.003	-0.014	<0.001	0.002	-0.029	<0.001	0.001
left insula	-0.049	0.012	0.023	-0.007	0.048	0.076	-0.023	0.004	0.008
right bankssts	-0.056	0.004	0.011	-0.005	0.197	0.239	-0.025	0.003	0.006
right caudalanteriorcingulate	-0.047	0.017	0.028	-0.003	0.506	0.538	-0.010	0.210	0.231
right caudalmiddlefrontal	-0.037	0.063	0.081	-0.008	0.046	0.074	-0.027	0.002	0.004
right cuneus	-0.022	0.268	0.285	-0.007	0.054	0.083	-0.004	0.623	0.632
right entorhinal	-0.067	0.001	0.003	-0.012	0.002	0.006	-0.015	0.069	0.091
right fusiform	-0.084	<0.001	<0.001	-0.013	0.001	0.005	-0.041	<0.001	<0.001
right inferiorparietal	-0.067	0.001	0.003	-0.018	<0.001	<0.001	-0.024	0.003	0.007
right inferiortemporal	-0.059	0.003	0.008	-0.012	0.002	0.006	-0.028	0.001	0.002
right isthmuscingulate	-0.051	0.010	0.022	-0.003	0.376	0.406	-0.019	0.019	0.029
right lateraloccipital	-0.042	0.034	0.050	-0.013	0.001	0.003	-0.021	0.007	0.013
right lateralorbitofrontal	-0.053	0.008	0.018	-0.006	0.113	0.154	-0.019	0.015	0.024
right lingual	-0.049	0.014	0.025	-0.006	0.096	0.135	-0.019	0.017	0.027
right medialorbitofrontal	-0.041	0.038	0.054	-0.005	0.191	0.239	-0.012	0.123	0.142
right middletemporal	-0.069	<0.001	0.002	-0.010	0.008	0.019	-0.030	<0.001	0.001
right parahippocampal	-0.051	0.010	0.022	-0.002	0.699	0.721	-0.025	0.002	0.004
right paracentral	-0.023	0.256	0.276	-0.006	0.145	0.185	-0.028	0.001	0.003
right parsopercularis	-0.044	0.025	0.036	-0.012	0.002	0.006	-0.018	0.026	0.038
right parsorbitalis	-0.030	0.125	0.146	-0.005	0.143	0.185	-0.013	0.094	0.114
right parstriangularis	-0.022	0.275	0.288	-0.007	0.081	0.117	-0.019	0.018	0.028
right pericalcarine	-0.025	0.198	0.224	-0.004	0.308	0.355	-0.017	0.042	0.058

right postcentral	-0.024	0.218	0.243	-0.010	0.013	0.026	-0.033	<0.001	<0.001
right posteriorcingulate	-0.081	<0.001	<0.001	-0.015	<0.001	0.001	-0.014	0.078	0.099
right precentral	-0.045	0.024	0.036	-0.010	0.020	0.037	-0.037	<0.001	<0.001
right precuneus	-0.068	0.001	0.002	-0.018	<0.001	<0.001	-0.030	<0.001	0.002
right rostralanteriorcingulate	0.007	0.707	0.707	<0.001	0.928	0.928	-0.003	0.726	0.726
right rostralmiddlefrontal	-0.047	0.017	0.028	-0.009	0.023	0.042	-0.015	0.054	0.073
right superiorfrontal	-0.050	0.011	0.022	-0.013	0.001	0.004	-0.030	<0.001	0.002
right superiorparietal	-0.050	0.012	0.023	-0.018	<0.001	<0.001	-0.024	0.004	0.007
right superiortemporal	-0.070	<0.001	0.002	-0.008	0.026	0.046	-0.032	<0.001	<0.001
right supramarginal	-0.091	<0.001	<0.001	-0.013	0.001	0.004	-0.022	0.007	0.013
right frontalpole	-0.047	0.017	0.028	-0.001	0.780	0.791	-0.019	0.015	0.024
right temporalpole	-0.089	<0.001	<0.001	-0.012	0.002	0.006	-0.023	0.004	0.009
right transversetemporal	-0.063	0.001	0.004	-0.012	0.002	0.006	-0.029	<0.001	0.002
right insula	-0.061	0.002	0.006	-0.010	0.011	0.025	-0.026	0.001	0.003

Table S12: Relationships between cortical surface area with clinical variables in PD

Region	HY disease stage			Time since diagnosis			Global cognition		
	ρ	P-val	P_{FDR}	β	P-val	P_{FDR}	β	P-val	P_{FDR}
left bankssts	-0.015	0.436	0.502	-0.007	0.060	0.203	-0.019	0.021	0.118
left caudalanteriorcingulate	-0.012	0.531	0.592	-0.003	0.358	0.603	-0.017	0.027	0.129
left caudalmiddlefrontal	-0.024	0.225	0.313	-0.006	0.127	0.287	-0.012	0.128	0.348
left cuneus	-0.048	0.015	0.051	-0.006	0.121	0.287	0.004	0.558	0.808
left entorhinal	-0.007	0.722	0.737	-0.004	0.348	0.603	-0.001	0.883	0.938
left fusiform	-0.057	0.004	0.025	-0.002	0.536	0.743	-0.003	0.745	0.872
left inferiorparietal	-0.041	0.038	0.088	-0.006	0.150	0.329	-0.017	0.036	0.144
left inferiortemporal	-0.063	0.002	0.015	-0.009	0.026	0.121	-0.002	0.833	0.914
left isthmuscingulate	-0.014	0.464	0.526	-0.001	0.854	0.908	-0.017	0.042	0.151
left lateraloccipital	-0.077	<0.001	0.002	-0.008	0.027	0.121	-0.006	0.456	0.756
left lateralorbitofrontal	-0.045	0.022	0.065	-0.011	0.004	0.054	-0.009	0.269	0.539
left lingual	-0.080	<0.001	0.002	-0.010	0.007	0.065	-0.005	0.494	0.800
left medialorbitofrontal	-0.062	0.002	0.015	-0.016	<0.001	0.002	-0.018	0.024	0.127
left middletemporal	-0.025	0.200	0.296	-0.008	0.025	0.121	-0.007	0.334	0.613
left parahippocampal	-0.016	0.406	0.476	-0.005	0.209	0.442	-0.016	0.042	0.151
left paracentral	-0.021	0.283	0.362	-0.002	0.534	0.743	-0.004	0.628	0.821
left parsopercularis	-0.026	0.181	0.280	0.003	0.372	0.603	-0.019	0.014	0.103
left parsorbitalis	-0.049	0.014	0.049	-0.006	0.121	0.287	-0.019	0.019	0.118
left parstriangularis	-0.028	0.152	0.247	<0.001	0.923	0.940	-0.017	0.030	0.137
left pericalcarine	-0.042	0.032	0.088	-0.008	0.029	0.121	0.004	0.611	0.821
left postcentral	-0.050	0.011	0.047	-0.001	0.805	0.899	-0.005	0.549	0.808
left posteriorcingulate	-0.037	0.063	0.129	-0.005	0.252	0.484	-0.026	0.001	0.024
left precentral	-0.024	0.225	0.313	<0.001	0.927	0.940	-0.013	0.095	0.281
left precuneus	-0.049	0.013	0.049	-0.003	0.444	0.643	-0.020	0.008	0.078
left rostralanteriorcingulate	<0.001	0.993	0.993	-0.003	0.372	0.603	-0.010	0.233	0.511
left rostralmiddlefrontal	-0.065	0.001	0.012	-0.014	<0.001	0.011	0.004	0.592	0.821

left superiorfrontal	-0.043	0.030	0.086	-0.007	0.081	0.252	<0.001	0.997	0.997
left superiorparietal	-0.056	0.005	0.026	-0.003	0.392	0.616	-0.024	0.001	0.023
left superiortemporal	-0.042	0.034	0.088	-0.003	0.404	0.616	-0.002	0.756	0.872
left supramarginal	-0.047	0.019	0.057	-0.006	0.127	0.287	-0.020	0.012	0.103
left frontalpole	-0.026	0.196	0.296	<0.001	0.905	0.940	-0.003	0.718	0.872
left temporalpole	-0.030	0.128	0.217	-0.001	0.807	0.899	-0.005	0.546	0.808
left transversetemporal	-0.011	0.573	0.628	0.002	0.569	0.773	-0.016	0.055	0.186
left insula	-0.010	0.623	0.672	0.004	0.322	0.591	0.002	0.825	0.914
right bankssts	-0.022	0.259	0.341	-0.005	0.237	0.475	-0.012	0.136	0.355
right caudalanteriorcingulate	-0.033	0.098	0.184	-0.009	0.018	0.114	-0.022	0.005	0.062
right caudalmiddlefrontal	-0.018	0.359	0.428	-0.002	0.597	0.781	-0.001	0.890	0.938
right cuneus	-0.057	0.004	0.025	-0.007	0.069	0.222	-0.001	0.924	0.938
right entorhinal	0.007	0.710	0.737	0.004	0.256	0.484	-0.004	0.617	0.821
right fusiform	-0.065	0.001	0.012	-0.008	0.058	0.203	-0.005	0.543	0.808
right inferiorparietal	-0.050	0.012	0.048	-0.006	0.095	0.270	-0.036	<0.001	<0.001
right inferiortemporal	-0.048	0.016	0.051	-0.003	0.437	0.643	-0.006	0.431	0.733
right isthmuscingulate	-0.008	0.698	0.737	0.001	0.770	0.899	-0.004	0.643	0.825
right lateraloccipital	-0.050	0.011	0.047	<0.001	0.966	0.966	-0.009	0.243	0.517
right lateralorbitofrontal	-0.076	<0.001	0.002	-0.009	0.012	0.089	-0.009	0.219	0.497
right lingual	-0.059	0.003	0.021	-0.006	0.086	0.254	-0.004	0.617	0.821
right medialorbitofrontal	-0.032	0.111	0.198	-0.009	0.018	0.114	-0.003	0.748	0.872
right middletemporal	-0.080	<0.001	0.002	-0.012	0.003	0.049	-0.009	0.252	0.519
right parahippocampal	-0.021	0.288	0.362	-0.007	0.109	0.286	-0.016	0.069	0.223
right paracentral	-0.019	0.341	0.414	-0.001	0.736	0.894	0.007	0.391	0.695
right parsopercularis	-0.038	0.055	0.117	0.003	0.371	0.603	-0.008	0.320	0.613
right parsorbitalis	-0.053	0.007	0.039	-0.009	0.021	0.117	-0.017	0.033	0.140
right parstriangularis	-0.024	0.226	0.313	-0.001	0.800	0.899	-0.013	0.121	0.344
right pericalcarine	-0.051	0.010	0.046	-0.010	0.005	0.057	0.002	0.829	0.914

right postcentral	-0.021	0.294	0.363	0.001	0.821	0.900	-0.007	0.399	0.695
right posteriorcingulate	-0.031	0.123	0.215	-0.006	0.107	0.286	-0.014	0.073	0.225
right precentral	-0.029	0.137	0.228	-0.001	0.762	0.899	-0.022	0.005	0.062
right precuneus	-0.036	0.068	0.137	-0.005	0.215	0.442	-0.019	0.020	0.118
right rostralanteriorcingulate	-0.032	0.110	0.198	-0.007	0.050	0.201	-0.011	0.178	0.417
right rostralmiddlefrontal	-0.041	0.037	0.088	-0.011	0.003	0.049	0.001	0.913	0.938
right superiorfrontal	-0.041	0.039	0.088	-0.008	0.054	0.203	-0.011	0.174	0.417
right superiorparietal	-0.041	0.037	0.088	-0.001	0.697	0.878	-0.026	0.001	0.023
right superiortemporal	-0.041	0.041	0.089	-0.010	0.011	0.089	-0.001	0.908	0.938
right supramarginal	-0.028	0.156	0.247	-0.002	0.581	0.775	-0.003	0.663	0.835
right frontalpole	-0.007	0.726	0.737	0.002	0.626	0.803	-0.007	0.332	0.613
right temporalpole	0.022	0.261	0.341	0.003	0.408	0.616	0.005	0.548	0.808
right transversetemporal	-0.035	0.079	0.154	-0.001	0.717	0.887	-0.011	0.162	0.408
right insula	-0.024	0.230	0.313	-0.001	0.850	0.908	0.003	0.730	0.872

Table S13: Relationships between subcortical volume with clinical variables in PD

Region	HY disease stage			Time since diagnosis			Global cognition		
	ρ	P-val	P_{FDR}	β	P-val	P_{FDR}	β	P-val	P_{FDR}
left lateral ventricle	0.088	<0.001	<0.001	0.004	0.361	0.413	0.039	<0.001	<0.001
left thalamus	-0.063	0.002	0.003	-0.012	0.002	0.005	-0.007	0.352	0.375
left caudate	-0.061	0.002	0.003	-0.019	<0.001	<0.001	-0.013	0.108	0.133
left putamen	-0.071	<0.001	0.001	-0.011	0.005	0.009	-0.036	<0.001	<0.001
left pallidum	-0.028	0.152	0.152	<0.001	0.982	0.982	-0.018	0.039	0.057
left hippocampus	-0.102	<0.001	<0.001	-0.015	<0.001	<0.001	-0.029	<0.001	0.001
left amygdala	-0.092	<0.001	<0.001	-0.023	<0.001	<0.001	-0.041	<0.001	<0.001
left accumbens	-0.061	0.002	0.003	-0.010	0.010	0.014	-0.028	0.001	0.001
right lateral ventricle	0.080	<0.001	<0.001	0.005	0.257	0.317	0.038	<0.001	<0.001
right thalamus	-0.047	0.018	0.021	-0.012	0.003	0.005	-0.007	0.412	0.412
right caudate	-0.059	0.003	0.003	-0.016	<0.001	<0.001	-0.015	0.067	0.089
right putamen	-0.061	0.002	0.003	-0.008	0.052	0.070	-0.023	0.005	0.007
right pallidum	-0.032	0.111	0.119	-0.002	0.580	0.619	-0.010	0.220	0.251
right hippocampus	-0.095	<0.001	<0.001	-0.016	<0.001	<0.001	-0.042	<0.001	<0.001
right amygdala	-0.082	<0.001	<0.001	-0.021	<0.001	<0.001	-0.036	<0.001	<0.001
right accumbens	-0.083	<0.001	<0.001	-0.011	0.005	0.009	-0.026	0.001	0.003

Table S14: Group-average node-neighbourhood correlations

Weighting	Network model	Correlation (ρ)	P value	$P_{spin/perm}$
structural connectivity	cortico-cortical	0.545	<0.001	0.001
	subcortico-cortical	0.187	0.523	0.495
functional connectivity	cortico-cortical	0.366	0.002	0.071
	subcortico-cortical	0.020	0.946	0.921

Table S15: Disease stage node-neighbourhood correlations

Weighting	Network model	HY stage	Correlation (ρ)	P value	$P_{spin/perm}$
structural connectivity	cortico-cortical	1	0.479	<0.001	0.004
		2	0.518	<0.001	0.003
		3	0.564	<0.001	0.001
		4/5	0.381	0.001	0.025
	subcortico-cortical	1	-0.204	0.483	0.478
		2	0.015	0.958	0.918
		3	0.209	0.474	0.449
		4/5	0.349	0.221	0.238
functional connectivity	cortico-cortical	1	0.516	<0.001	0.006
		2	0.327	0.007	0.104
		3	0.387	0.001	0.056
		4/5	0.228	0.061	0.258
	subcortico-cortical	1	-0.385	0.175	0.165
		2	-0.077	0.794	0.806
		3	0.130	0.659	0.679
		4/5	0.112	0.703	0.671

Table S16: Neurotransmitter system annotation enrichment of cortical thickness

Neurotransmitter system	Receptor/transporter	Correlation (ρ)	P_{spin}	P_{FDR}
serotonin	5HT1a	-0.178	0.504	0.605
	5HT1b	0.493	0.002	0.018
	5HT2a	-0.095	0.619	0.619
	5HT4	-0.340	0.069	0.177
	5HT6	0.197	0.099	0.191
	5HTT	0.289	0.066	0.177
acetylcholine	A4B2	0.503	0.004	0.024
	M1	0.190	0.328	0.470
	VAChT	0.119	0.369	0.474
cannabinoid	CB1	-0.237	0.340	0.470
dopamine	D1	0.095	0.601	0.619
	D2	0.263	0.098	0.191
	DAT	0.537	0.001	0.018
GABA	GABAa	0.073	0.612	0.619
histamine	H3	0.187	0.147	0.240
glutamate	mGluR5	0.298	0.106	0.191
opioid	MOR	0.367	0.026	0.094
norepinephrine	NET	0.322	0.007	0.031

Table S17: Neurotransmitter system annotation enrichment of cortical surface area

Neurotransmitter system	Receptor/transporter	Correlation (ρ)	P_{spin}	P_{FDR}
serotonin	5HT1a	0.195	0.316	0.517
	5HT1b	-0.074	0.750	0.831
	5HT2a	-0.287	0.082	0.184
	5HT4	-0.082	0.501	0.694
	5HT6	0.006	0.831	0.831
	5HTT	0.304	0.078	0.184
acetylcholine	A4B2	0.358	0.012	0.054
	M1	0.044	0.750	0.831
	VAChT	0.174	0.437	0.655
cannabinoid	CB1	0.099	0.823	0.831
dopamine	D1	0.397	0.028	0.101
	D2	-0.005	0.801	0.831
	DAT	0.343	0.012	0.054
GABA	GABAa	-0.325	0.010	0.054
histamine	H3	0.258	0.126	0.252
glutamate	mGluR5	0.181	0.205	0.369
opioid	MOR	0.329	0.036	0.108
norepinephrine	NET	0.595	0.001	0.018

Table S18: Neurotransmitter system annotation enrichment of subcortical volume

Neurotransmitter system	Receptor/transporter	Correlation (ρ)	P_{perm}	P_{FDR}
serotonin	5HT1a	-0.662	0.009	0.081
	5HT1b	-0.055	0.847	0.953
	5HT2a	-0.569	0.040	0.126
	5HT4	-0.244	0.442	0.759
	5HT6	-0.262	0.361	0.721
	5HTT	0.130	0.656	0.844
acetylcholine	A4B2	0.552	0.042	0.126
	M1	-0.464	0.099	0.241
	VAChT	-0.169	0.552	0.770
cannabinoid	CB1	-0.002	0.991	0.991
dopamine	D1	0.073	0.798	0.953
	D2	-0.688	0.009	0.081
	DAT	-0.165	0.556	0.770
GABA	GABAa	-0.627	0.016	0.096
histamine	H3	-0.596	0.023	0.103
glutamate	mGluR5	0.209	0.464	0.759
opioid	MOR	0.451	0.107	0.241
norepinephrine	NET	0.011	0.946	0.991

Table S19: Summary of gene set enrichment analysis of biological processes for PLS1

Gene Set	Description	Size	Leading Edge Number	ES	NES	P-val	P_{FDR}
GO:0099003	vesicle-mediated transport in synapse	205	96	-0.456	-2.305	<0.001	<0.001
GO:0035249	synaptic transmission, glutamatergic	97	36	-0.431	-1.921	<0.001	0.045
GO:0048499	synaptic vesicle membrane organization	27	10	-0.548	-1.921	<0.001	0.031
GO:0051648	vesicle localization	203	73	-0.374	-1.874	<0.001	0.044
GO:0006813	potassium ion transport	191	69	-0.352	-1.744	<0.001	0.142
GO:0034728	nucleosome organization	61	22	-0.421	-1.730	0.004	0.137
GO:0016358	dendrite development	217	64	-0.337	-1.703	<0.001	0.147
GO:0099177	regulation of trans-synaptic signaling	425	125	-0.305	-1.673	<0.001	0.135
GO:0050803	regulation of synapse structure or activity	225	66	-0.330	-1.668	<0.001	0.128
GO:0048013	ephrin receptor signaling pathway	46	17	-0.428	-1.654	0.010	0.134
GO:1990868	response to chemokine	56	27	0.481	1.975	<0.001	0.007
GO:0045785	positive regulation of cell adhesion	361	131	0.371	2.003	<0.001	0.006
GO:0007229	integrin-mediated signaling pathway	91	39	0.445	2.005	<0.001	0.007
GO:0050886	endocrine process	59	33	0.480	2.016	<0.001	0.006
GO:0002347	response to tumor cell	34	19	0.549	2.034	<0.001	0.004
GO:0060840	artery development	85	31	0.466	2.092	<0.001	0.002
GO:0060976	coronary vasculature development	40	16	0.550	2.095	<0.001	0.003
GO:0019882	antigen processing and presentation	92	45	0.493	2.232	<0.001	<0.001
GO:0002181	cytoplasmic translation	148	90	0.469	2.262	<0.001	<0.001
GO:0002396	MHC protein complex assembly	18	13	0.733	2.280	<0.001	<0.001

ES = enrichment score; NES = normalized enrichment score.

Table S20: Summary of gene set enrichment analysis of cellular components for PLS1

Gene Set	Description	Size	Leading Edge Number	ES	NES	P-val	P_{FDR}
GO:0060076	excitatory synapse	62	21	-0.510	-2.068	<0.001	0.009
GO:0098978	glutamatergic synapse	390	136	-0.359	-1.952	<0.001	0.012
GO:0044298	cell body membrane	29	15	-0.535	-1.879	0.004	0.019
GO:0097060	synaptic membrane	354	145	-0.350	-1.877	<0.001	0.014
GO:0030427	site of polarized growth	153	48	-0.375	-1.849	<0.001	0.016
GO:0043198	dendritic shaft	40	16	-0.498	-1.849	<0.001	0.013
GO:0044304	main axon	60	20	-0.441	-1.826	<0.001	0.013
GO:0098984	neuron to neuron synapse	339	104	-0.333	-1.788	<0.001	0.019
GO:0044309	neuron spine	165	55	-0.365	-1.786	<0.001	0.018
GO:0099572	postsynaptic specialization	320	135	-0.334	-1.778	<0.001	0.018
GO:0005775	vacuolar lumen	139	63	0.321	1.554	0.002	0.096
GO:0101031	protein folding chaperone complex	40	21	0.431	1.607	0.019	0.077
GO:0005796	Golgi lumen	65	34	0.408	1.718	0.002	0.031
GO:0072562	blood microparticle	71	36	0.415	1.779	<0.001	0.021
GO:0005840	ribosome	230	116	0.386	1.982	<0.001	0.001
GO:0098636	protein complex involved in cell adhesion	39	19	0.531	2.019	<0.001	0.001
GO:0009897	external side of plasma membrane	237	98	0.393	2.042	<0.001	0.001
GO:0062023	collagen-containing extracellular matrix	298	129	0.392	2.073	<0.001	0.001
GO:0098576	lumenal side of membrane	34	17	0.656	2.379	<0.001	<0.001
GO:0042611	MHC protein complex	21	17	0.818	2.608	<0.001	<0.001

ES = enrichment score; NES = normalized enrichment score.

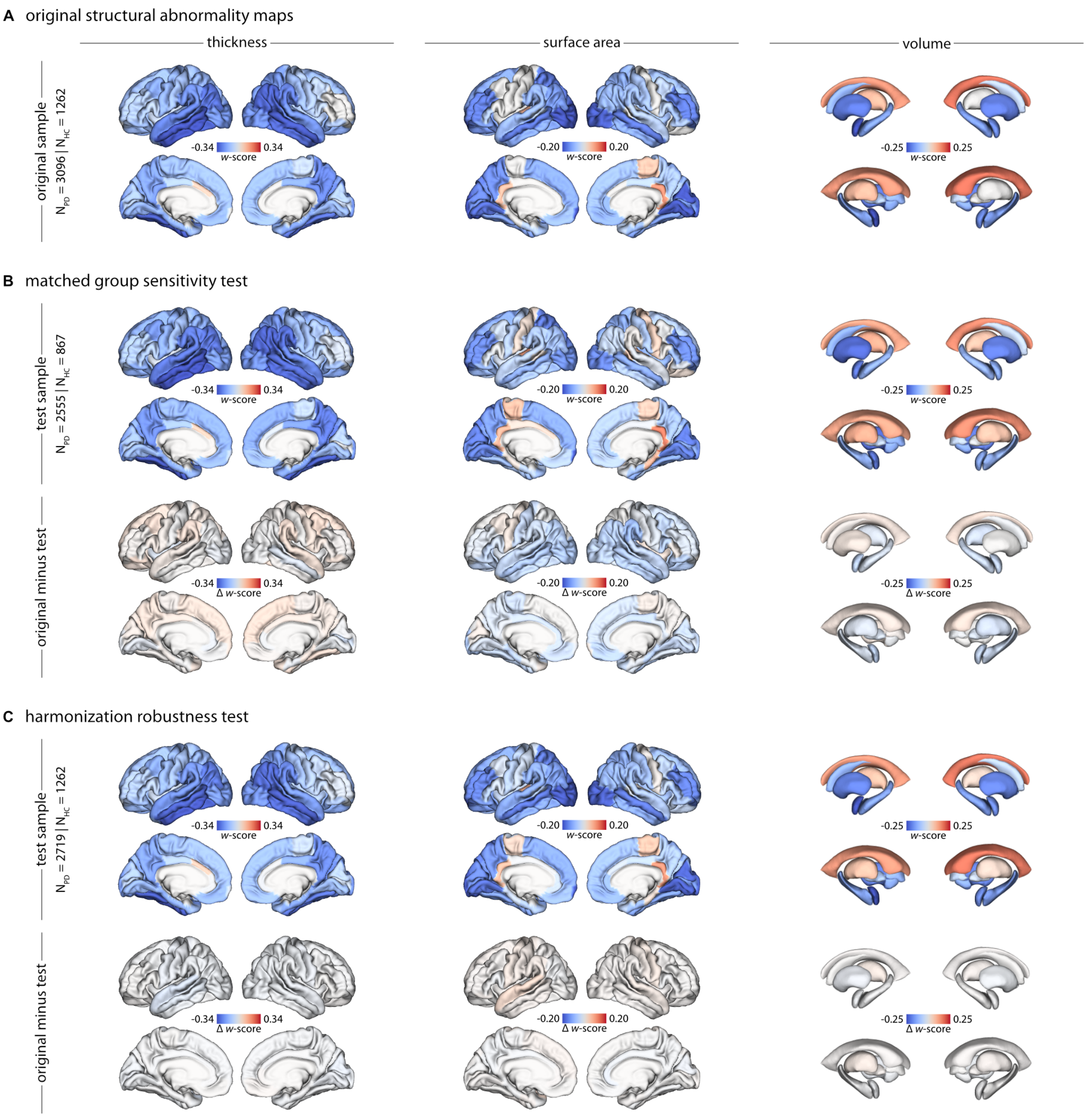

Figure S1: *W*-score maps of cortical thickness, cortical surface area, and subcortical volume from **(A)** the original study sample, **(B)** a test subsample after Hoehn and Yahr stage stratified, propensity score matching with replacement before computing *w*-scores, and **(C)** a test subsample after cohorts comprised of only PD patients, but no HCs were removed before harmonization. *W*-score difference maps between the original study sample and **(B)** the matched group test subsample or **(C)** the harmonization test subsample.

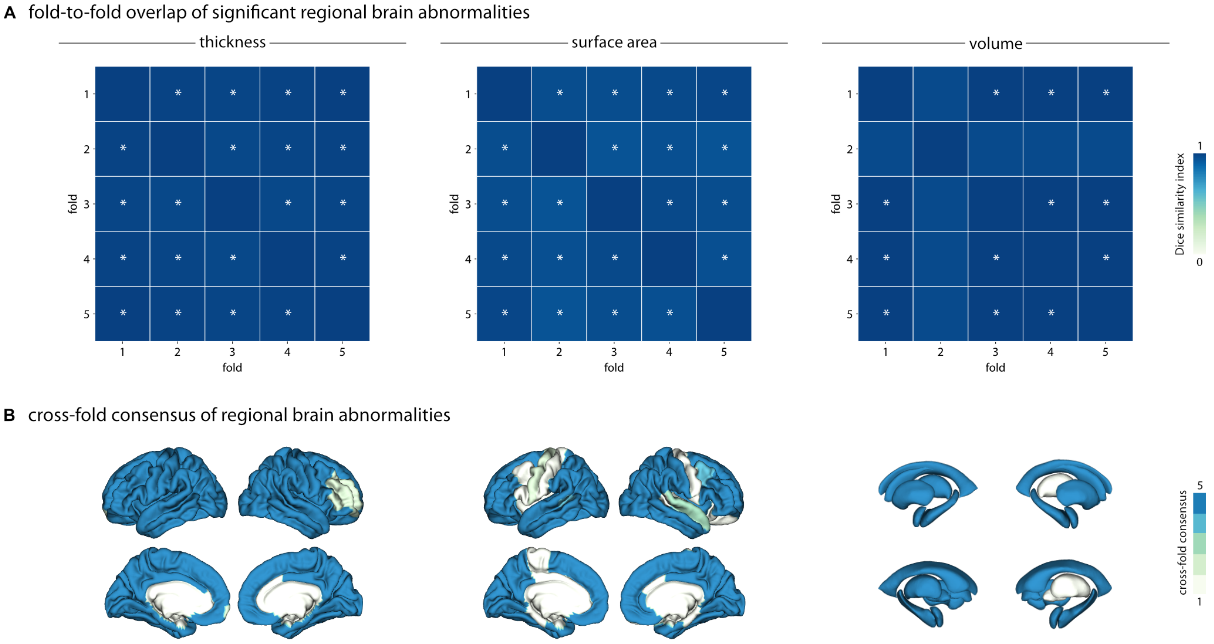

Figure S2: N-fold cross-validation of cortical thickness, cortical surface area, and subcortical volume *w*-scores in PD demonstrated the robustness and consistency of the brain atrophy pattern in PD across different subsets of the sample. For each fold (n = 5), a stratified subset of PD patients (preserving age, sex, and disease stage distributions of the full sample) was withheld and the remaining data were used to generate binarized maps of significant regional deviations in PD ($P_{FDR} < 0.05$). **(A)** Pairwise Dice similarity indices and permutation testing (n = 1,000) revealed high cross-fold consistency of brain regions with significant regional deviations. **(B)** A high degree of cross-fold consensus was observed across most brain regions.

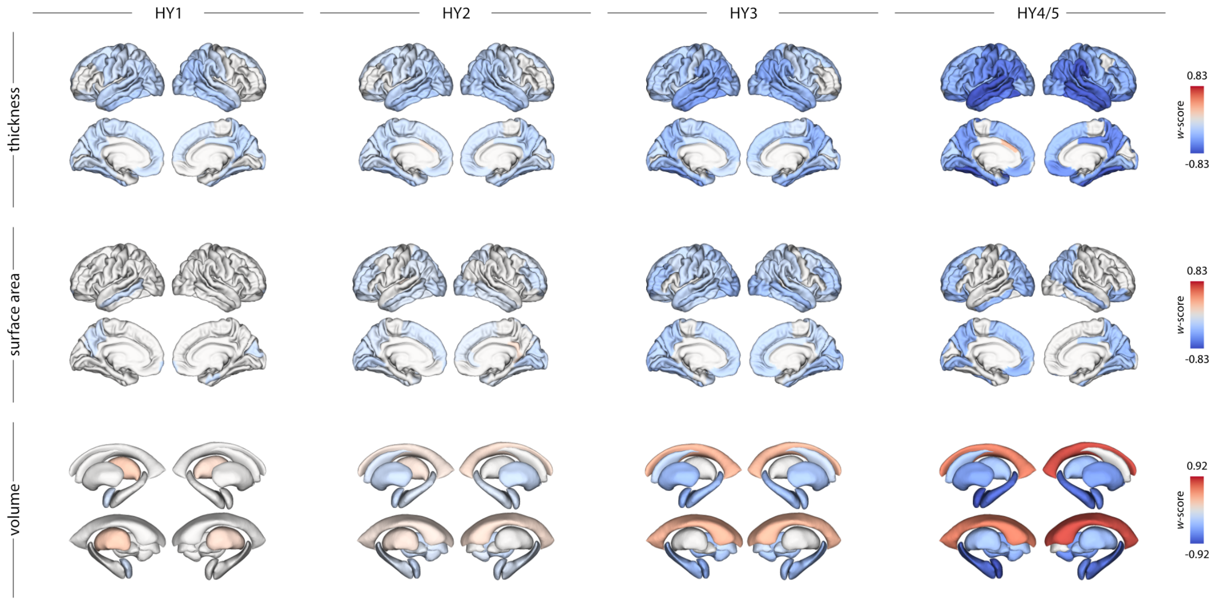

Figure S3: *W*-score maps of cortical thickness (top row), surface area (middle row), and subcortical volume (bottom row) across Hoehn and Yahr disease stages (left to right). *W*-scores are plotted using a shared colour scale for each brain measure to allow visual comparisons across disease stages. Only regions surviving false discovery rate correction for multiple comparisons (P_{FDR} < 0.05) are displayed.

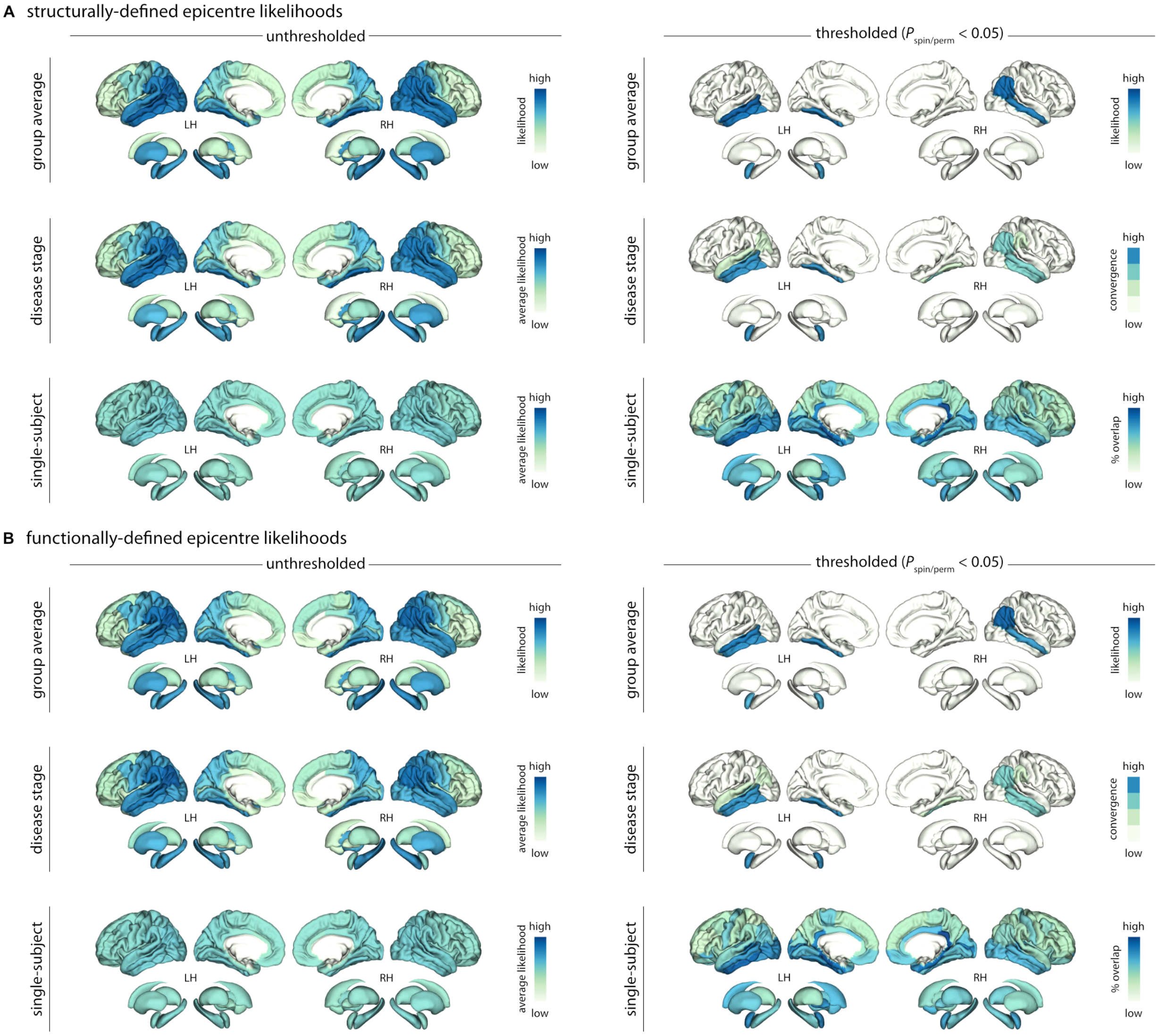

Figure S4: Brain maps of **(A)** structurally- and **(B)** functionally-defined epicentre likelihoods. Group average level (top rows) displays epicentre likelihood ranks (left) and significant epicentres (right; $P_{\text{spin/perm}} < 0.05$) for the group average atrophy map. Disease stage level (middle rows) shows average epicentre likelihood ranks (left) and convergence of significant epicentres ($P_{\text{spin/perm}} < 0.05$) across the four Hoehn and Yahr disease stages. Single-subject level (bottom rows) shows average epicentre likelihood ranks (left) and convergence of significant epicentres ($P_{\text{spin/perm}} < 0.05$) across individual atrophy maps.

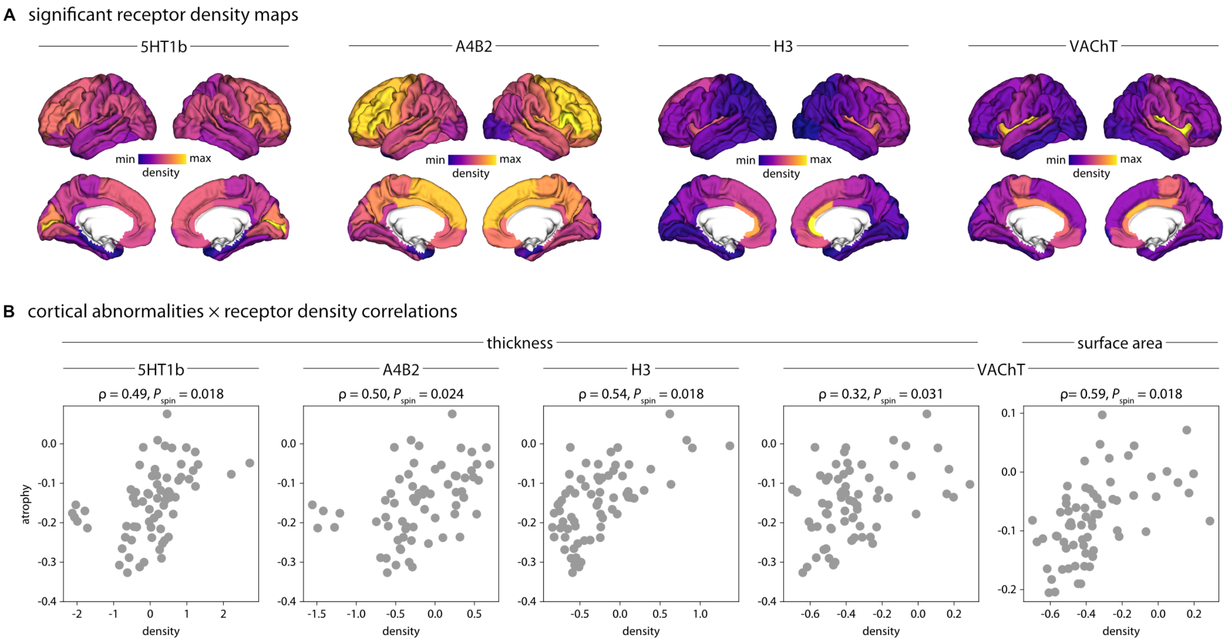

Figure S5: **(A)** Surface maps of neurotransmitter receptor densities significantly correlated with the pattern of cortical atrophy in PD. **(B)** Scatter plots of significant correlations between neurotransmitter receptor densities and cortical atrophy. 5-HT1B = serotonin; α4β2 = nicotinic acetylcholine receptor; H3 = histamine; VAChT = vesicular acetylcholine transporter

Structural and functional MRI data

Structural and functional connectivity networks were obtained from the *enigmatoolbox*[1], derived from diffusion-weighted imaging and resting-state functional MRI data in a cohort of unrelated healthy adults from the Human Connectome Project (n = 207, 83 males, mean age ± SD = 28.74 ± 3.73 years, range = 22-36 years).[2] Diffusion-weighted imaging data (spin-echo EPI sequence, TR = 5520 ms, TE = 89.5 ms, FOV = 210 × 180, voxel size = 1.25 mm^3, *b*-value = 1000/2000/3000 s/mm^2, 270 diffusion directions, 18 b0 images) underwent b0 intensity normalization and were corrected for susceptibility distortion, eddy currents, and head motion. Resting-state functional MRI data (gradient-echo EPI sequence, TR = 720 ms, TE = 33.1 ms, FOV = 208 × 180 mm^2, voxel size = 2 mm^3, 72 slices) were corrected for distortion, head motion, and magnetic field bias, and underwent skull removal, intensity normalization, and registration to MNI152 space.[3] Automatic removal of noise components (e.g., head motion, white matter, cardiac pulsation, arterial, and large vein-related effects) was performed using FSL FIX.[4] The resulting preprocessed time series were transformed into grey matter ordinate space using cortical ribbon-constrained volume-to-surface mapping and combined into a single time series.

1. Larivière S, Paquola C, Park B yong, et al. The ENIGMA Toolbox: multiscale neural contextualization of multisite neuroimaging datasets. *Nat Methods*. 2021;18(7):698-700. doi:10.1038/s41592-021-01186-4
2. Elam JS, Glasser MF, Harms MP, et al. The Human Connectome Project: A retrospective. *NeuroImage*. 2021;244:118543. doi:10.1016/j.neuroimage.2021.118543
3. Glasser MF, Sotiropoulos SN, Wilson JA, et al. The minimal preprocessing pipelines for the Human Connectome Project. NeuroImage. 2013;80:105-124. doi:10.1016/j.neuroimage.2013.04.127
4. Salimi-Khorshidi G, Douaud G, Beckmann CF, Glasser MF, Griffanti L, Smith SM. Automatic denoising of functional MRI data: Combining independent component analysis and hierarchical fusion of classifiers. NeuroImage. 2014;90:449-468. doi:10.1016/j.neuroimage.2013.11.046
